# Mediterranean Diet: Prevention of Colorectal Cancer

**DOI:** 10.3389/fnut.2017.00059

**Published:** 2017-12-05

**Authors:** Micah G. Donovan, Ornella I. Selmin, Tom C. Doetschman, Donato F. Romagnolo

**Affiliations:** ^1^Department of Nutritional Sciences, University of Arizona, Tucson, AZ, United States; ^2^University of Arizona Cancer Center, Tucson, AZ, United States; ^3^Department of Molecular and Cellular Medicine, University of Arizona, Tucson, AZ, United States

**Keywords:** nutrition, carcinogenesis, inflammatory bowel diseases, epigenetics, microbiome

## Abstract

Colorectal cancer (CRC) is the third most common cancer diagnosis and the second and third leading cause of cancer mortality in men and women, respectively. However, the majority of CRC cases are the result of sporadic tumorigenesis *via* the adenoma–carcinoma sequence. This process can take up to 20 years, suggesting an important window of opportunity exists for prevention such as switching toward healthier dietary patterns. The Mediterranean diet (MD) is a dietary pattern associated with various health benefits including protection against cardiovascular disease, diabetes, obesity, and various cancers. In this article, we review publications available in the PubMed database within the last 10 years that report on the impact of a MD eating pattern on prevention of CRC. To assist the reader with interpretation of the results and discussion, we first introduce indexes and scoring systems commonly used to experimentally determine adherence to a MD, followed by a brief introduction of the influence of the MD pattern on inflammatory bowel disease, which predisposes to CRC. Finally, we discuss key biological mechanisms through which specific bioactive food components commonly present in the MD are proposed to prevent or delay the development of CRC. We close with a discussion of future research frontiers in CRC prevention with particular reference to the role of epigenetic mechanisms and microbiome related to the MD eating pattern.

## Introduction

Despite advancements in screening and diagnosis, colorectal cancer (CRC) remains the third most common cancer in the USA and the second and third leading cause of cancer mortality in men and women, respectively (1). Interestingly, only ~5–6% of CRC cases are linked to germline mutations (2), whereas ~70% of CRC tumors are sporadic (3). These statistics suggest great opportunities may exist for the prevention of CRC. The initiation and progression through the adenoma–carcinoma sequence is multifactorial and influenced by a variety of environmental factors (4). Depending on the eating pattern, diet may ameliorate or even increase CRC risk (5).

**Figure 1:**
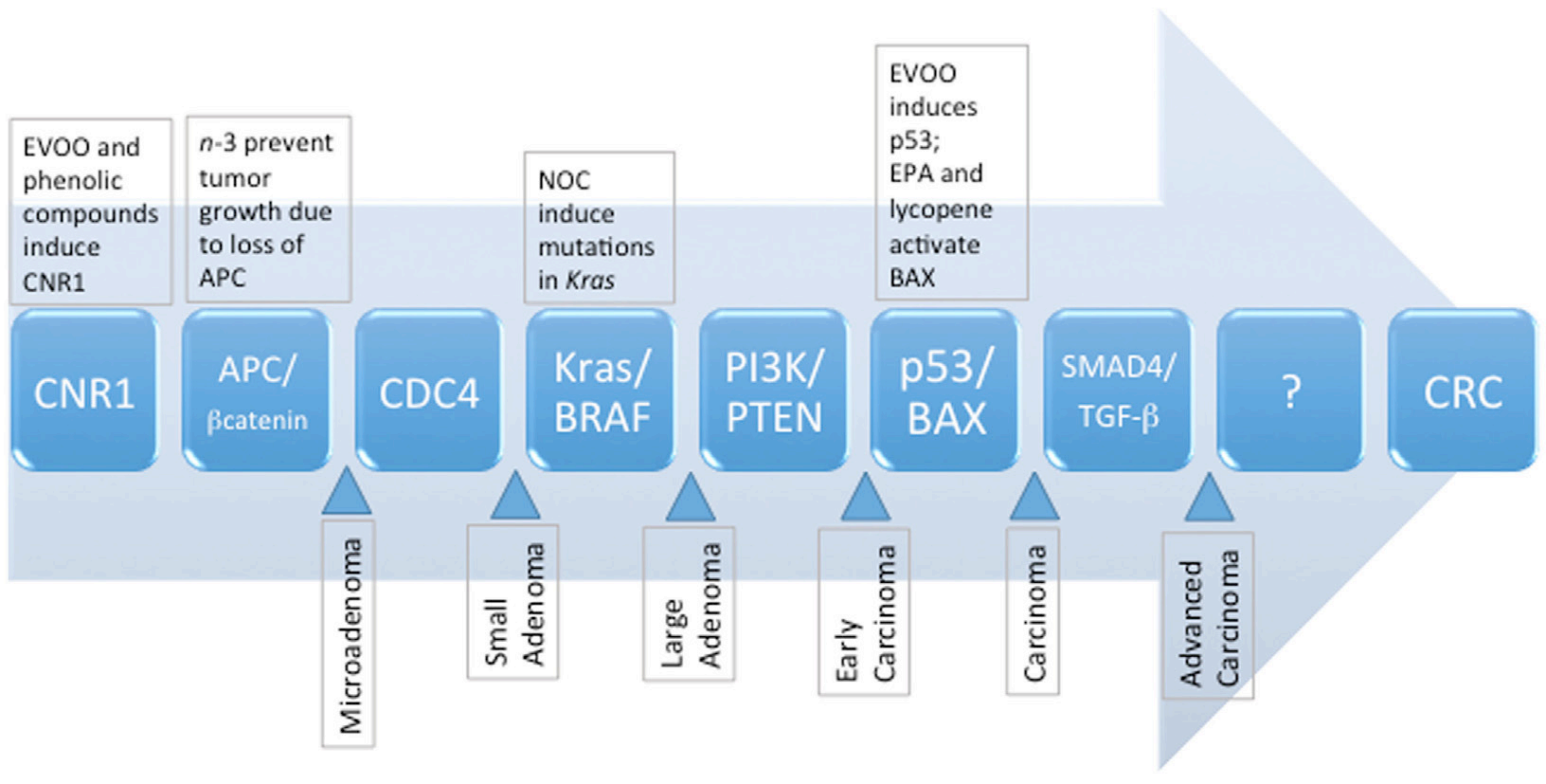
The sequence of known genetic mutations that accumulate and manifest as metastatic colorectal cancer, and the potential dietary influences of selected compounds commonly present in the MD pattern. EPA, eicosapentaenoic acid; EVOO, extravirgin olive oil; NOC, *N*-nitroso compounds, MD, Mediterranean diet.

For an in-depth analysis of genetic aberrations associated with the development of CRC, we direct the reader to excellent reviews by Mundade et al. (6), Amaro et al. (7), and Mármol et al. (3). Briefly, three major molecular pathways of sporadic CRC have been identified, and include (1) chromosomal instability (CIN), characterized by abnormal karyotypes, aneuploidy, and loss of heterozygosity; (2) microsatellite instability (MSI), characterized by silencing of DNA repair mechanisms, namely mismatch repair pathways; and (3) CpG island methylator phenotype (CIMP), associated with hypermethylation and silencing of tumor suppressor genes (3, 6). Recent estimates indicate that ~24% of CRC carry the MSI or CIMP phenotype; and ~27% have MSI and CIN (8). The CIN pathway, also known as the adenoma–carcinoma sequence (Figure 1), appears to be predominantly involved in ~80–85% of sporadic CRC (9). Mechanisms contributing to the CIN phenotype include telomere dysfunction and alterations in chromosome segregation, and DNA damage, leading to altered expression of genes including *APC, KRAS, PI3K*, and *TP53* (3). Loss of *APC* leads to increased nuclear transcriptional activity of β-catenin and prolonged activation of the Wnt pathway (10). Normally, β-catenin is sequestered and degraded in the cytosol by a complex comprising APC and other accessory proteins. However, the sustained activity of β-catenin results in the development of a stem cell phenotype. Cells do not migrate to the epithelial surface to be sloughed off in the intestinal lumen, leading to the accumulation of undifferentiated cells in colonic crypts and polyp formation. Progression through the adenoma to carcinoma sequence is further driven by *KRAS* and *PI3K* mutations, and constitutive activation of the mitogen-activated protein (MAP) kinase (MAPK) pathway, accompanied by uncontrolled cell cycle entry due to mutations in *TP53* (3).

Progression through the various stages of the adenoma–carcinoma sequence takes ~15–20 years, whereas the transition from carcinomas to metastatic tumors involves markedly less time (~2–3 years) (11). The relatively extended period of time required for progression through the benign stages of the adenoma–carcinoma sequence suggests environmental factors, such as the diet, significantly influence CRC development. The health benefits of the Mediterranean diet (MD) have been extensively documented in the literature and include protection against cardiovascular disease, diabetes, obesity, and various cancers. This is also reflected in its inclusion as a recommended dietary pattern, among others (i.e., Healthy US Style Eating Pattern and Healthy Vegetarian Eating Pattern) in the 2015 Dietary Guidelines for Americans (12). This raises the question whether adherence to a MD offers protection against the development of CRC. To this end, we have reviewed preclinical and clinical studies investigating the effect of the MD or its components on CRC tumorigenesis. We precede the review of research evidence with a short description of the MD eating pattern; various indexes and scores used to estimate adherence to the MD pattern; and how foods and bioactive components in the MD may protect from chronic intestinal inflammation, such as that seen in inflammatory bowel disease (IBD), a condition that predisposes to CRC. We follow-up our review with a brief discussion of the role of the MD in epigenetic processes and changes in intestinal microbiota related to CRC.

## MD Eating Pattern

The MD eating pattern refers to dietary behaviors established through the blending of food, religious, economic and cultural practices by civilizations that have occupied the Mediterranean basin for millennia (13). Twenty-two countries located across three continents are considered to have contributed to the MD eating pattern. As a result, there is no one absolute definition for the MD. Instead, this pattern can be described as one that (1) is abundant in plant-based foods such as whole grains, legumes, nuts, seeds, fruits, and vegetables; (2) comprises olive oil as the main source of dietary fat; (3) limits intakes of red and processed meat, saturated fat, and refined sugars; (4) favors low-to-moderate intake of low-fat dairy and moderate consumption of fish; and (5) emphasizes regular, but moderate, alcohol (mostly red wine) consumption with meals (14). The MD eating pattern also recommends the inclusion of water, tea, and herbal infusions as non-alcoholic beverages (13, 15, 16).

Several components of the MD such as olive oil, fish, red wine, tea, fruits and vegetables, and other fiber sources contribute a myriad of bioactive compounds including antioxidants (i.e., proanthocyanidins, flavonoids, and other polyphenolic compounds); *n*-3 polyunsaturated fatty acids (PUFAs) such as eicosapentaenoic acid (EPA), docosahexaenoic acid (DHA); and short-chain fatty acids (SCFAs; i.e., butyrate). Given the diversity of available foods and dietary practices among Mediterranean countries, various definitions of the MD have been proposed such as one that favors higher intake of monounsaturated (MUFA) over saturated (SFA) fatty acid (MUFA/SFA ~1.6–2.0); dietary fiber (41–62 g/day); antioxidants (~3,500–5,300 6-hydroxy-2,5,7,8-tetramethylchroman-2-carboxylic acid equivalent/day); and phytosterols (~370–555 mg/day) (17). The MD has been extensively studied over several decades and suggested to provide protection against various chronic diseases and various cancers, including CRC (18, 19).

### MD Scores and Indexes

A recent review identified a total of 22 Mediterranean index and score systems differing in the number of components, scoring range, and type of food components (20). A detailed discussion of these indexes is beyond the scope of this work. Therefore, we refer the reader to the description of the Mediterranean pyramid as reported by the Mediterranean Foundation Group (21). However, we provide a short description of some MD score systems (Table 1; Data Sheet S1 in Supplementary Material) and their potential usefulness to predict adherence to a MD eating pattern.

**Table 1:** Summary of Mediterranean diet scoring systems applied in reviewed studies.

System	Index components	Scoring^a^	Differences from MDS
MDS	Alcohol, cereals, dairy, fish, fruits and nuts, legumes, meat and meat products, MUFA:SFA ratio, vegetables^b^	Range: 0–9	N/A^c^
		One point for intake *at or greater than* study-specific median of non-bold items	
		One point for intake *less than* study-specific median of bold items	
		One point for alcohol intake of 5–25 g/day for women and 10–50 g/day for men (otherwise 0)	

aMED	Alcohol, fish, fruit, legumes, MUFA:SFA^d^, nuts, red and processed meat, vegetables^e^, whole grains	Range: 0–9	Potatoes excluded from vegetable group Fruits and nuts separated into 2 groups Eliminated dairy group Included group for all whole grains Only red and processed meat in meat group Modified range for alcohol intake
		One point for intake *at or greater than* study-specific median of non-bold items	
		One point for intake *less than* study-specific median of bold items.	
		One point for alcohol intake of 5–15 g/day	

IMDI	Alcohol, butter, fish, fruit, legumes, Mediterranean vegetables, olive oil, pasta, potatoes, red meat, soft drinks	Range: 0–11	Wider scoring range Points awarded for intake w/in third tertile rather than ≥median Potatoes excluded from vegetables and assigned its own group Included individual groups for butter, olive oil, pasta, and soft drinks Excluded group for cereals and whole grains (pasta included instead) Modified range for alcohol intake
		One point for intake within third tertile of study distribution of non-bold items	
		One point for intake within first tertile for bold items.	
		One point for alcohol intake up to 12 g/day, 0 points for >12 g/day and no intake	

MMDS	Alcohol, cereals, dairy, fish, fruits and nuts, legumes, lipid ratio^f^, meat and meat products, vegetables	Range: 0–9	Addition of PUFA to numerator of lipid ratio (PUFA + MUFA/SFA = lipid ratio)
		One point for intake *at or greater than* study-specific median of non-bold items	
		One point for intake *less than* study-specific median of bold items	
		One point for alcohol intake of 5–25 g/day for women and 10–50 g/day for men (otherwise 0)	

*^a^Component assigned score of 0 if intake does not meet criteria for 1 point*.

*^b^Includes potatoes*.

*^c^Original MDS used to compare changes introduced with newer systems*.

*^d^MUFA:SFA ratio defined as monounsaturated fatty acid intake divided by saturated fatty acid intake*.

*^e^Excludes potatoes*.

*^f^Lipid ratio defined as polyunsaturated fatty acid plus monounsaturated fatty acid intake divided by saturated fatty acid intake (PUFA + MUFA)/SFA*.

The MD Score (MDS) considers the consumption of nine different foods including non-refined cereals, dairy, fish, fruits and nuts, legumes, meat and meat products, MUFA/SFA, vegetables (including potatoes), and alcohol (22). The total MDS ranges from “0” to “9” with higher scores indicating closer adherence to the MD eating pattern. The alternate Mediterranean diet (aMED) scoring system (0–9) was developed by Fung et al. (23) as an adaptation to the MDS. The aMED system considers the intake of fish, fruit, legumes, MUFA/SFA, nuts, red and processed meats, vegetables (excluding potatoes), and whole grains. The Italian MD index (IMDI) was developed by Agnoli et al. (24) as an adaptation of the Greek MD index. This system provides a score based on intake levels across 11 items including butter, fish, fruit, legumes, Mediterranean vegetables, olive oil, pasta, potatoes, red meat, soft drinks, and alcohol (24). Finally, the modified MD score (MMDS) was designed (25) to assess the intake across nine food components including those considered beneficial (vegetables, legumes, fruits, cereals, fish) and detrimental (meat and dairy products), and ethanol intake. In general, these systems are in good accord when considering fruits and vegetables as healthy, and meats as negative, dietary choices. However, some systems differ with regard to criteria for estimating moderate alcohol consumption as well as cut off values of intake (i.e., medians, tertiles, or established servings) (26). In general, MD indexes have shown satisfactory performance in assessing adherence to this dietary pattern. However, further studies are needed to better account for MD pattern variations (20).

## MD Eating Pattern and Inflammation-Related CRC

Chronic intestinal inflammation, such as that seen in Crohn’s disease (CD) and ulcerative colitis (UC) is a predisposing condition to CRC (27). This is demonstrated by the increased risk of CRC seen in IBD patients (28, 29). Colorectal carcinogenesis in IBD is believed to be similar to the adenoma–carcinoma sequence of sporadic CRC (30). Several of the genetic alterations observed in sporadic colorectal carcinogenesis are observed in colitis-associated CRC (31, 32). However, differences in the timing and frequency of genetic alterations have been observed. For example, loss of APC occurs with less frequency and at a later stage in IBD-related CRC and loss of p53 occurs earlier and in nondysplastic tissue of IBD-related CRC patients (30). In addition, the upregulation of proinflammatory factors such as cyclooxygenase-2 (COX2) is observed in IBD-related CRC. The capacity for the MD to prevent against CRC is likely due in part to the sum of anti-inflammatory effects exerted by the various food components that contribute to this dietary pattern, particularly those foods and beverages contributing a significant load of phenolic compounds (i.e., olive and fish oil, and plant-based foods). Although an extensive review of the effects of bioactive compounds commonly present in the MD on the etiology of IBD is beyond the scope of this work, we briefly highlight some of the overall findings pointing to protection by major components of the MD from biological end points of colonic and systemic inflammation. The information presented in this section has been summarized in Table 2.

**Table 2:** Summary of studies investigating anti-inflammatory effects of MD components.

Study type/dietary component	Model/population	Treatment	Biological outcome	Reference
**Preclinical studies**				

***n*-3 fatty acids**				
	HT29 and Caco2 cells	1:1 EPA/DHA (v. untreated control)	↓ COX-2	(33)
	DSS-rats	2:1 LA/ALA (v. 10:1)	↓ TNFα, IL-1β, MPO, ALP (colon)	(34)
	DSS-mice	2:1 LA/ALA (v. 4:1)	↓ TNFα, IL-17 (colon)	(35)
	TNBS-rats	*n*-3-enriched diet (30% total fat)	Improved HIS	(36)
			↓ PGE2, LTB4 (colon)	
	IL-10(−/−) mice	DHA supplemented diet (35.5 mg/kg/day)	Improved HIS	(37)
			↓ TNFα, IL-17 (colon)	
			↓ Inflammatory cell infiltration (colon)	
	IL-10(−/−) mice	EPA-enriched diet (3.7% by weight)	↓ Bacterial-induced inflammation	(38)

**Fiber**				
	HLA-B27 rats	High-fiber diet (5% psyllium seed by weight, 13 weeks)	↓ TNFα, LTB4, NO (colon)	(39)
	HLA-B27 rats	High-fiber diet (5 g/kg/day, 7 weeks)	↓ IL-1β (cecal tissue)	(40)
			↑ TGFβ (cecal tissue)	
	TNBS-rats	Lactulose-enriched water (2.5% wt/vol, 2 weeks)	↓ TNFα, LTB4 (colonic mucosa)	(41)
	DSS-rats	Lactulose (300–1,000 mg/kg twice daily, 6 days)	↓TNFα, LTB4 (cecal tissue)	(42)
	DSS-mice	Butyrate-enriched diet (0.5% by weight)	↓ IL-10 (colon)	(44)
			↓ Presence of dendritic cells and memory T lymphocytes in colon	
			↑ TGFβ (colon)	
	IL-10(−/−) mice	Butyrate supplement (100 mg/kg/day)	Improved HIS	(45)
			↓ NF-κB signaling	

**Olive oil**				
	HT29 and Caco2 cells	200 µM/l EVOO	↓ COX-2	(33)
	DSS-rats	EVOO-enriched diet (5%)	Improved DAI, HIS	(48)
			↓ COX-2, iNOS, STAT3 (colonic mucosa)	
	DSS-mice	EVOO-enriched diet (10%)	Improved DAI, HIS	(49)
			↓ COX-2, iNOS, TNFα, IL-10 (colon)	
	DSS-mice	EVOO unsaponifiable fraction	Improved DAI, HIS	(50)
			↓ COX-2, iNOS, TNFα, MCP1	
	IL-10(−/−) mice	Olive oil-enriched diet (7% by weight)	↓ COX-2 (colon)	(51)
			↓ Colitis-associated neoplasia	

**Phenolic compounds**				
	PG-PS-rats	Resveratrol (100 mg/kg, postPG-PS injection)	↓ IL-1β, IL-6, TNFα, and TGFβ (cecal tissue)	(52)
	DSS-mice	Resveratrol (3 mg/kg BW)	↓ COX-2, iNOS, TNFα, and IL-1β (colon)	(53)
			↑ IL-10 (colon)	
	IL-10(−/−) mice	Resveratrol (100 mg/kg BW)	Improved clinical scores	(54)
			↓ TNFα, IL-6, IL-12, IL-1β (colon)	
	TNBS-rats	Resveratrol (10 mg/kg diet)	↓ PGE2, COX-2, and NF-κB (colon)	(55)

**Clinical studies**				

***n*-3 fatty acids**				
	Overweight males (BMI > 25)	EPA (58 mg/day) + DHA (32.5 mg/day) (4 weeks)	↓ IL-1β, IL-6, TNFα	(62)
			↑ Adiponectin (circulating)	
	Male and female MS patients (on interferon therapy)	EPA (0.8 g/day) + DHA (1.6 g/day) (1 year)	↓ TNFα, IL-1β, IL-6, NO metabolites (circulating)	(63)
	Male and female UC patients (on prednisone)	EPA (3.24 g/day) + DHA (2.16 g/day) (4 months)	Improved HIS	(65)
			↓ LTB4 (rectal)	
			↓ Avg. needed dose of prednisone	
	Male and female UC patients	EPA (3.2 g/day) + DHA (2.4 g/day) (6 months)	↓ IL-2 (circulating)	(66)
			↓ NK cell cytotoxicity	

**Fiber**				
	Male and female UC patients	20–30 g Germinated barley fiber supplement	Improved endoscopic index parameters and clinical activity	(68)

**Olive oil**				
	Spanish coronary heart disease patients	50 ml/day EVOO (2 × 3 weeks periods)	↓ IL-6, CRP (circulating)	(70)
	Young (20–30) and old (65–85) healthy subjects	25 ml/day EVOO (12 weeks)	↑ HDL and PON11 anti-inflammatory capacity	(71)

*ALP, alkaline phosphatase; COX-2, cyclooxygenase-2; CRP, C-reactive protein; DAI, disease activity index; HDL, high-density lipoprotein; HIS, histological index scores; IL, interleukin; iNOS, inducible nitric oxide synthase; LTB4, leukotriene B4; MCP-1, monocyte chemoattractant protein-1; MPO, myeloperoxidase; NF-κB, nuclear factor kappa-light-chain-enhancer of activated B cells; NK, natural killer; NO, nitric oxide; PGE2, prostaglandin E2; PON1, paraoxonase 1; STAT, signal transducer and activator of transcription; TGF-β, transforming growth factor beta; TNFα, tumor necrosis factor alpha*.

## Preclinical Studies on Anti-Inflammatory Effects of Bioactive MD Components

### *n*-3 Fats

Studies in CRC cells demonstrated that a fish oil mixture (1:1 ratio of EPA and DHA) decreased expression of the COX-2 gene (*PTSG2*) (33). In experiments using the dextran sodium sulfate (DSS)-induced UC rat model, a diet with a 2:1 ratio of *n*-6 linoleic acid (LA) to *n*-3 α-linolenic acid (ALA) improved clinical activity and decreased colonic levels of several proinflammatory factors such as tumor necrosis factor-alpha (TNFα) and interleukin (IL)-1β (34). Similarly, in DSS-mice a diet rich in *n*-3 fatty acids (2:1 LA/ALA) decreased colonic TNFα and IL-17 (35). Experiments utilizing the trinitrobenzene sulfonic acid (TNBS)-induced UC rat model demonstrated an *n*-3-enriched diet (30% of total fat) improved histological index scores (HIS) and decreased alkaline phosphatase and γ-glutamyltranspeptidase activity, in addition to decreasing colonic levels of proinflammatory factors (36). In IL-10 deficient [IL-10(−/−)] mice, DHA (i.e., 35.5 mg/kg/day) decreased colonic infiltration of inflammatory cells, improved HIS scores, and decreased expression of proinflammatory cytokines (i.e., IL-17, TNFα) (37). These effects were observed in parallel with enhanced barrier function. Another study in IL-10(−/−) mice reported EPA (3.7% by weight) to be protective against bacterial-induced inflammation and this effect was attributed to peroxisome proliferator-activated receptor-alpha (PPARα) signaling (38).

### Fiber

Studies in the HLA-B27 transgenic rat model of CD showed a high fiber diet (5% psyllium seed by weight) decreased colonic levels of nitric oxide, LTB4, and TNFα (39). Another study in HLA-B27 rats on a high fiber diet (5 g/kg body weight/day) demonstrated decreased IL-1β expression in cecal tissue and improved HISs (40). In female TNBS-rats, dietary supplementation with lactulose through drinking water (2.5% wt/vol) reduced TNFα and LTB4 levels in colon tissue (41). Decreased levels of cecal TNFα and leukotrienes were also observed in male DSS-rats given oral lactulose (300–1,000 mg/kg BW) twice daily (42).

Butyrate, a SCFA byproduct of bacterial fermentation in the gut, is proposed to contribute to the oncoprotective effects of fiber, given that it decreased cell proliferation (40–100 mM) and cell migration (10–50 mM), and induced apoptosis (10–50 mM) in human RKO colon cancer cells (43). A butyrate-enriched diet (0.5% by weight) decreased TGF-β, but increased IL-10, memory T lymphocytes and dendritic cells in intestinal mucosa of DSS-mice (44). In another study, administration of oral butyrate (100 mg/kg) was also reported to improve HIS in both IL-10(−/−) mice and DSS-mice (45). The authors of this study attributed these benefits of butyrate to inhibition of NF-κB signaling and histone deacetylation.

### Olive Oil and Phenolic Compounds

Two major components of olive oil are 18:1 oleic acid (~70%) and phenolic compounds [i.e., tyrosol, hydroxytyrosol, catechin, epicatechin, epigallocatechin gallate (EGCG), oleuropein, quercetin, and rutin] (46, 47). *In vitro* studies attributed the anti-inflammatory effect of olive oil to the phenolic fraction, rather than oleic acid. In colon cancer cells, whole olive oil, but not oleic acid, downregulated expression of COX-2 (33). This suggests that phenolic compounds, rather than oleic acid content alone, should be considered when evaluating the health benefits of different olive oil preparations. Extra virgin olive oil (EVOO) (5% enriched diet) administered to DSS-rats improved disease activity indexes (DAIs) and HIS, and decreased expression of several inflammatory factors in colonic tissue (48). In DSS-mice, an EVOO-enriched diet (10%) showed similar protective effects in regard to DAI, HIS and colonic levels of proinflammatory markers (49). Additional anti-inflammatory effects were observed upon dietary supplementation with EVOO plus hydroxytyrosol confirming the important role of phenolic compounds in olive oil (49). Other studies in DSS mice identified the unsaponifiable fraction of olive oil was efficacious in improving DAI and HIS in addition to decreasing colonic expression of monocyte chemoattractant protein-1 (MCP-1) and various proinflammatory factors (i.e., TNFα; COX-2, iNOS) (50). An anti-inflammatory effect of olive oil has also been demonstrated in genetic models of IBD. For example, in IL-10(−/−) mice, olive oil-supplemented chow (7%) decreased colonic COX-2 levels and reduced the risk of chronic colitis-related neoplasia (51).

Polyphenols found in grapes and red wine may also reduce gut inflammation. In the peptidoglycan-polysaccharide (PG-PS) rat model of CD, resveratrol (100 mg/kg, administered post-PG-PS injection) decreased cecal expression of IL-1β, IL-6, TNFα, and TGFβ (52). In DSS-mice, daily supplementation with resveratrol (3 mg/kg BW) decreased COX-2, iNOS, TNFα, and IL-1β and increased expression of anti-inflammatory IL-10 (53). Studies using the IL-10(−/−) mouse model demonstrated administration of resveratrol by oral gavage (100 µl of 10, 50, or 100 mg/kg) improved clinical scores and decreased colonic levels of several inflammatory markers including TNFα, IL-6, IL-12, and IL-1β (54). The anti-inflammatory effects of resveratrol in the colon have also been reported in TNBS-rats (55). Overall, these preclinical studies support the idea that consumption of bioactives commonly present in the MD may be useful for the prevention of biological end points associated with colonic inflammation.

## Clinical Studies on the Influence of Diet in Systemic Inflammation and IBD

### Whole Diet

An observational study deriving data from the 1936 Lothian birth cohort documented that adopting a MD reduced serum levels of fibrinogen, a biomarker of systemic inflammation (56). Another study in a cohort from the Athenian province of Attica reported reduced C-reactive protein (CRP), fibrinogen, IL-6, TNFα, and homocysteine levels were associated with high compliance to MDS over a 10-year period (57). A meta-analysis of 17 randomized controlled trials evaluated the impact of the MD on development of IBD (58). Overall, results of this meta-analysis suggested that adoption of a MD eating pattern decreased biomarkers associated with systemic inflammation (high sensitivity-CRP) and endothelial dysfunction [intercellular adhesion molecule-1 (ICAM-1)] (58).

A crossover trial compared the effects of a MUFA-rich MD to those of a SFA-rich or low-fat-high-carbohydrate (LFHC) diet on the postprandial inflammatory state of elderly men and women from Córdoba Spain (mean age 67.1 years) (59). At the end of a 3-week intervention period, subjects on the MD had decreased fasting and postprandial expression of p65, a subunit of NF-κB, in isolated peripheral blood mononuclear cells (PBMCs), compared to subjects on the SFA-rich diet. Also, subjects on the MD diet had decreased postprandial expression of p65 and TNFα compared to subjects on the LFHC diet (59). Another study in Italian men and women with metabolic syndrome (MetS) (mean age 43 years) examined the effects of adherence to a MD on markers of systemic inflammation (60). At the end of the 2-year intervention period, subjects following the MD eating pattern had higher levels of high-density lipoprotein (HDL) and lower serum levels of IL-6, IL-7, IL-18, and hs-CRP compared to controls (60).

In CD patients, the anti-inflammatory action of a MD that included salmon, organic avocados, kumara (a variety of sweet potato), gluten-free bread, EVOO, green tea, honey, and fish oil has been investigated using a transcriptomic approach (61). Findings suggested that adoption of the MD eating pattern resulted in upregulation of ~1,900, and downregulation of ~1,650 genes. After 6 weeks of adherence to the MD, there was a trend for reduced serum levels of CRP and DNA damage in PBMC (61). Further characterization of these gene expression and biochemical changes is crucial to develop molecular markers that predict the health benefits of the MD eating pattern against IBD.

### *n*-3 Fats

An intervention trial in overweight males [body max index (BMI) > 25, 25–65 years of age] found daily supplementation with ~58 mg of EPA and 32.5 mg of DHA for 4 weeks decreased circulating levels of proinflammatory IL-1β, IL-6, and TNFα, while increasing circulating levels of adiponectin (62). Similar results were observed in another study providing 0.8 g EPA plus 1.6 g DHA/day in a Mexican population with multiple sclerosis (18–55 years) currently undergoing interferon therapy (63). After 1 year of intervention, subjects that supplemented their diet with fish oils had markedly reduced levels of serum TNFα, IL-1β, IL-6, and NO metabolites compared to control subjects (63). These results suggested that the anti-inflammatory benefits of *n*-3 fatty acids commonly present in the MD eating pattern could be extended to non-Mediterranean groups. This idea has been tested in a prospective study in the United Kingdom and showed a decreased risk of UC in association with increased intake of DHA, and borderline significant lower risk with EPA and total *n-*3 intake (64). Other studies noted that in UC patients taking prednisone, supplementation with EPA (3.24 g/day) and DHA (2.16 g/day) for 4 months lowered rectal LTB4 levels; improved acute and total HIS; and decreased the average needed dose of prednisone (65). Parallel studies also showed that supplementation with EPA (3.2 g/day) plus DHA (2.4 g/day) for 6 months reduced natural killer cell cytotoxicity and serum IL-2 in UC patients (66). Overall, these clinical studies support the conclusion that *n*-3 fatty acids present in fish oil, namely EPA and DHA, protect against development of IBD.

### Fiber

A cross-sectional study of healthy subjects from the Italian cohort of the European Prospective Investigation into Cancer and Nutrition (EPIC) (67) reported an inverse relationship between intake of fiber and circulating levels of various markers of systemic inflammation (i.e., IL-1β, IL-4, IL-5, IL-6, IL-13, and TNFα) (67). A clinical trial with UC patients showed a reduction in endoscopic index parameters (erythema, edema, friability, granularity, erosion) and clinical activity (e.g., diarrhea, nocturnal diarrhea, blood in stools, incontinence, abdominal pain, or cramping) as a result of supplementation with fiber (20–30 g/day of germinated barley) (68).

### Olive Oil

Epidemiological data suggest that olive oil, in particular EVOO, is effective for the prevention of systemic inflammatory responses (69). In Spanish coronary heart disease patients, doses of 50 ml/day of unprocessed EVOO over two 3-week periods reduced serum levels of IL-6 and CRP compared to control subjects receiving refined olive oil (70). Another study showed olive oil (25 ml/day for 12 weeks) increased the anti-inflammatory activity of HDL and paraoxonase 1 (PON1) (71). In *ex vivo* experiments using patient-derived samples, EA.hy926 endothelial hybrid cells had significantly decreased expression of ICAM-1 when cocultured with HDL extracted from subjects receiving olive oil, compared to cocultures containing HDL from control subjects (71). Taken together, results of these preclinical and clinical studies suggest consumption of foods commonly found in the MD eating pattern attenuates symptoms associated with systemic and gastrointestinal inflammation, a condition that predisposes to CRC.

## MD Eating Pattern and CRC

### Methods

The main objective of this review was to interrogate the PubMed database for studies that reported on the association between adherence to a MD eating pattern and risk of CRC. We began with the search criteria “Mediterranean diet AND colorectal cancer” with selection limited to clinical studies published during the past 10 years. We then performed secondary screening of preclinical and clinical studies investigating specific food components including cereals and/or grains, vegetables, fruit, legumes, fish and/or fish oil and/or *n-3* fatty acid, olive oil, alcohol, red and/or processed meat, potatoes, butter, and sugar and/or sweets. An example query for a secondary search was: “Mediterranean vegetables AND colorectal cancer” or “Mediterranean dairy AND colorectal cancer.” Studies included in this review were primary preclinical or clinical investigations examining the association between the MD and risk of CRC. Studies were included if adherence to the MD was assessed using a validated index (i.e., MDS) or a dietary pattern that coincided with traditional MD parameters. We included studies that investigated individual foods or food components only if they were performed in the context of the MD (i.e., in a Mediterranean population). We excluded reviews or systematic reviews without a meta-analysis, and dietary studies of CRC that did not focus on a MD eating pattern. Initial search queries generated 223 publications. After filtering by inclusion/exclusion criteria, we identified 40 studies for review.

### Preclinical Studies of CRC

#### Sugars

Emphasis on intake of non-refined plant-based foods may improve insulin resistance and decrease circulating levels of insulin-like growth factor-1 (IGF-1), a proproliferative and antiapoptotic growth factor (72). Although receptors for both insulin and IGF-1 are expressed in normal colorectal cells, high circulating levels of these growth factors support malignant transformation (73). Indeed, preclinical studies showed that a combination of insulin and IGF-1 promoted cell cycle progression and proliferation of murine colon cancer MC38 cells *in vitro via* activation of extracellular signal-regulated kinases (ERK)1/2 and c-Jun N-terminal kinase (JNK)/MAPK signaling (74). IGF-1 has also been shown to enhance the growth of MC38 colon cancer allografts in rodent models (75). Therefore, reducing the intake of foods that stimulate circulating and intestinal IGF-1 production may help in reducing the risk of CRC.

#### Meat and Meat Products

Consumption of red and processed meat has been linked to increased risk of CRC through production of mutagenic *N*-nitroso compounds (NOCs) in the gastric environment (76). Approximately 50% of CRC are known to harbor mutated KRAS, namely a G > A transition at codons 12 or 13, which are characteristic of NOC-mediated DNA alkylation (77). NOCs have also been shown to induce DNA deamination leading to DNA polymerase and mismatch errors, and the formation of abasic sites (78). Evidence suggests guanine residues are more sensitive to *N*-nitroso deamination than adenine and cytosine (78). Cooking of meat at high temperatures generates heterocyclic amines (HCA) and polycyclic aromatic hydrocarbons (PAH) (79, 80). Both HCA and PAH have been shown to be carcinogenic in animal studies and associate with the development of various tumor types in humans (81).

#### Fruits and Vegetables

Compounds in fruits and vegetables characteristic of the MD may protect against development of CRC. For example, 3,3′-diindolylmethane (72) and sulforaphane (73) from cruciferous vegetables (i.e., broccoli, Brussels sprouts, kale) were found to inhibit growth of colon cancer cells through cell cycle arrest. Anthocyanin-rich berry extracts (74) and procyanidins (75) present in red wine, teas, olive oil, and various fruits (i.e., apples, grapes, currants) were also shown to inhibit growth of colorectal (Caco2) cancer cells. In HCT-8 CRC cells, the compound (3S)-1,2,3,4-tetrahydro-β-carboline-3-carboxylic acid derived from chicory root increased apoptosis associated with activation of caspases (-3, -8, and -9) (82) and B-cell lymphoma-2 (BCL-2)-4 [Bcl-2-associated X protein (BAX)]; and downregulation of BCL-2 and NF-κB (82). Ginger leaf extracts were also found to reduce cell viability and increase apoptosis in HCT-116, SW480, and LoVo CRC cells *via* ERK-dependent upregulation of activating transcription factor 3 (83). In rodent models, dietary supplementation with scallion extracts antagonized growth of CT-26 colon cancer xenografts and enhanced survival rate compared to control-fed animals (84). Molecular effects attributed to the scallion extract included inhibition of proinflammatory COX-2 and iNOS; proliferative cyclin D1 and cellular myelocytomatosis (c-MYC); angiogenic vascular endothelial growth factor and hypoxia inducible factor-1α; and invasion matrix metallopeptidase-9 and ICAM-1 factors.

#### Olive Oil

Results of several preclinical studies support a protective action of olive oil against CRC. For example, pretreatment of human HT29 colon cancer cells with a phenolic-rich virgin olive oil extract was shown to markedly reduce hydrogen peroxide-mediated DNA damage (85). Also, the phenol-rich extract improved Caco2 cell barrier function and decreased HT115 cell invasion (85). In human RKO and HCT116 colon cancer cells, treatment with pinoresinol-rich olive oil exerted, respectively, p53- and BAX-dependent antiproliferative and proapoptotic effects; while inducing ATM-dependent G2/M arrest (86). In DLD1 colon cancer cells, hydroxytyrosol decreased antioxidant defense capacity resulting in decreased cell proliferation and increased apoptosis, while attenuating forkhead box O3 (FOXO3) transcriptional activation of target genes *via* phosphatidylinositol-4,5-bisphosphate 3-kinase (PI3K)/protein kinase B (AKT) activation (87). Interestingly, hydroxytyrosol did not appear to affect normal colon epithelial cell growth, suggesting that the antiproliferative effect of this compound may be specific to transformed cells (87). The unsaponifiable fraction of olive oil, which consists of squalene, triterpenic or aliphatic alcohols, sterols, tocopherols, and other compounds has also demonstrated anti-CRC properties. In HT29 colon cancer cells, the treatment with the unsaponifiable fraction decreased proliferation and increased apoptosis paralleled by upregulation of p53 and PPARγ; downregulation of COX-2; and accumulation of nuclear factor of kappa light polypeptide gene enhancer in B-cells inhibitor-α (IKBα), an inhibitor of proinflammatory NF-κB (88).

Another study reported an olive olive-enriched diet (12% olive oil) suppressed intestinal polyp growth in male APC^Min/+^ mice (89). After 10 weeks, mice on the olive oil diet had significantly less polyps and polyp volume compared to control-fed animals (12% soybean oil). In contrast to control-fed mice, there was no difference between polyp and healthy tissue proliferative activity in mice on the olive oil diet. Growth inhibition of intestinal polyps was attributed to increased apoptotic activity. These effects were associated with decreased phosphorylation of signal transducer and activator of transcription 3 (STAT3), which is known to induce antiapoptotic genes, and an increased ratio of BAX/Bcl-2. Investigation of estrogen receptor (ER) expression demonstrated an increased ratio of ERβ/ERα in mice on the olive oil diet. Despite high sequence homology to ERα (>60%) (90), ERβ activation is associated with inhibition of cell growth and is thought to act as a negative regulator of estrogenic drive (91). The effects of olive oil in the study utilizing the male APC^Min/+^ mice demonstrated similar effects with an *n-3* fatty acid-rich diet (12% salmon oil) with respect to polyp number and volume, proliferative and apoptotic activity, and ER expression (89).

#### *n*-3 Fish Oils

In preclinical studies, findings about influence of *n-3* PUFA on development of CRC remain inconclusive. For example, a DHA-enriched diet (3%) administered for 7 weeks to adenomatous popyposis Coli (*Apc*) knockout female mice (*Apc*^Δ716^), decreased polyp multiplicity (92). However, no effects were seen in *Apc*^Δ716^ males. This sex-specific effect was also observed in *Apc^Min/+^* mice (*Apc* mutated), in which a fish oil-enriched diet (0.4, 1.25, or 2.5% of 54.4% EPA/30.3% DHA blend) decreased precancerous polyp multiplicity in females but not in males (93). Conversely, studies with male *Apc^Min/+^* mice administered an EPA-rich diet (2.5 or 5% EPA) reported decreased polyp size and multiplicity compared to male mice fed a control diet (94). Also, in male *Apc^Min/+^* mice, an EPA-rich diet (31 g/kg) was found to reduce tumor multiplicity (~50%) compared to animal controls (95). Similarly, in male Wistar rats, administration of fish-oil-rich diet (18% by weight for 36 weeks) was found to attenuate 1,2-dimethylhydrazine-induced preneoplastic lesion and adenoma development compared to control animals given a soybean rich diet (96). The reasons behind these apparently contrasting sex-related results remain largely unknown. Perhaps, interactions between gender and duration and level of intake may influence the cancer response to fish oils. Discrepancy between studies may also be attributed to between-study variation with respect to the use of different transgenic models (i.e., *Apc*^Δ716^ vs. *APC^Min/+^*) and dietary exposures (i.e., DHA only vs. ethyl esters of DHA and EPA, etc.). Interestingly, *in vitro* experiments suggested synergistic effects between fish oil and intake of other MD food components (i.e., tomatoes) (97). Combined treatment with EPA and lycopene, a bioactive compound derived from tomatoes, synergistically decreased HT-29 colon cancer cell proliferation. The latter effect was attributed to suppression of PI3K/AKT and mammalian target of rapamycin signaling, in addition to upregulation of the proapoptotic factors BAX and Fas ligand (97). Thus, studies are needed to clarify the gender-specific effects of fish oils and modifying effects of other key bioactive compounds present in the MD.

### Clinical Studies of CRC

#### Whole Diet and Colorectal Adenoma

Cottet et al. (89) utilized cohort data from the European Cancer Prevention study to examine the association between dietary pattern and risk of colorectal adenoma recurrence. Applying principle components analysis to dietary intake data, investigators identified three distinct dietary patterns: (1) a MD pattern, characterized by high consumption of olive oil, fresh fruit, vegetables, legumes, lean meat and fresh fish intake and low consumption of coffee, meat, beer, fats, whole-grain bread and cereals, and delicatessen; (2) a Western dietary pattern, with high intake of potatoes, fats, delicatessen products, high-fat meat, beer, rice, pasta, refined bread and cereals, nuts, and sodas; and (3) a snacks dietary pattern, with high positive loading for high-fat delicatessen meats, high-fat cheese, desserts and sweets, beer, soda, and mineral water. Multiple logistic regression models adjusted for age, treatment group, and presence of multiple and proximal adenomas at inclusion indicated a decreased risk of CR adenoma recurrence associated with the MD pattern in women. No other dietary pattern was associated with risk for women, and none of the patterns displayed an association in men.

A case–control study (*n* = 564 cases, 1,202 endoscopy negative controls) examined the association between a MD or Paleolithic style diet (PD) pattern and incidence of sporadic colorectal adenomas in a population residing in Minneapolis, St. Paul, MN, USA (98). Adherence to the MD was established with the MDS and a PD was described to have diverse and high intakes of vegetables, fruits, lean meats, fish, nuts, and calcium; and low intakes of red and processed meat, sodium, dairy, grains and starches, baked goods, sugar sweetened beverages, and alcoholic beverages. Both patterns displayed a significant linear trend toward decreased risk across all tertiles (MD, *P*_trend_ = 0.05; PD, *P*_trend_ = 0.02). Separate analyses by sex revealed a decreased risk for both dietary scores for men, but no association was noted for women possibly due to differences in mean age between cancer cases (58.1 ± 9.7) and controls (46.5 ± 6.4).

#### Whole Diet and CRC

##### Case Control

Table 3 summarizes three case–control studies reported in PubMed and documenting decreased odds of CRC associated with MD adherence. A study conducted in Athens, Greece examined the effect of the MD on CRC risk in the presence of metabolic MetS (73). Among 250 patients with no previous cancer diagnosis (mean average = 63 ± 12 years), and 250 age–gender-matched controls, MMDS scores were associated with decreased odds of CRC with 12% lower risk/unit increase in MMDS (*P* < 0.001). Investigators stratified data based on presence (MetS+) or absence of (MetS−) of MetS, and noted a 16 and 11% decrease in odds of CRC for every unit increase in MMDS score for MetS+ and MetS− subjects, respectively (73).

**Table 3:** Summary of epidemiological studies investigating association between MD adherence and colorectal cancer risk.

Design	Reference	Cohort/population	Sex	Age	Model	Adjustment	Effect	HR/OR/RR (95% CI)
**Case–control**								
	Kontou et al. (73)	Athens, Greece	M/W	NR	MMDS	Age, BMI, family Hx, PA, sex, smoking	Decreased risk	OR = 0.88 (0.84–0.92)
	Grosso et al. (99)	Catania, Italy	M/W	65.3^a^	MDS	Age, alcohol intake, diabetes, family Hx, obesity, PA, sex, smoking	Decreased risk	OR = 0.46 (0.28–0.75)
	Rosato et al. (100)	Various Italian cities^b^	M/W	19–74	MDS	Age, BMI, calendar period, center, education, energy intake, family Hx, PA	Decreased risk	OR = 0.52 (0.43–0.62)
**Cohort**								
	Reedy et al. (101)	NIH-AARP	M/W	50–71	MDS	Age, BMI, education, energy intake, PA, race, smoking, HRT (women only).	Decreased risk (M) No effect (W)	RR = 0.72 (0.63–0.83) RR = 0.89 (0.72–1.11)
	Fung et al. (103)	NHS and HPFS	M/W	30–75	aMED	Age, alcohol intake, BMI, colonscopy, energy intake, family Hx, multivitamin use, no. of polyps, NSAID use, PA, smoking	No effect No effect (M) No effect (W)	RR = 0.89 (0.77–1.01) RR = 0.88 (0.71–1.09) RR = 0.88 (0.74–1.05)
	Agnoli et al. (24)	Italian EPIC cohort	M/W	25–70	IMDI	Age, BMI, education, energy intake, PA, sex, smoking	Decreased risk Decreased risk (M) Decreased risk (W)	HR = 0.50 (0.35–0.71) HR = 0.54 (0.30–0.96) HR = 0.46 (0.30–0.72)
	Bamia et al. (102)	EPIC	M/W	25–70	MMDS CS-MMDS	Age, BMI, education, PA, sex, smoking	Decreased risk No effect (M) Decreased risk (W) Decreased risk No effect (M) No effect (W)	HR = 0.89 (0.90–0.99) HR = 0.89 (0.76–1.04) HR = 0.88 (0.77–1.01) HR = 0.92 (0.84–1.00) HR = 0.91 (0.80–1.03) HR = 0.93 (0.83–1.05)
	Vargas et al. (104)	WHI	W^c^	50–79	aMED	Age, education, PA, race, smoking, HRT	No effect	HR = 0.91 (0.74–1.11)

*^a^Population mean*.

*^b^Milan, Genoa, Pordenone, Gorizia, Forli, Latina, Naples, and Udine*.

*^c^Postmenopausal women*.

*AARP, American Association EPIC, European Prospective Investigation into Cancer and Nutrition; M, men; NHS, Nurses’ Health Study; NIH-AARP, National Institutes of Health Diet and Health Study; NR, not reported; W, women; WHI, Women’s Health Initiative*.

The MDS has been applied to a population from Catania, Italy (*n* = 338 cases of first CRC diagnosis, *n* = 676 matched healthy controls) to evaluate the association between lifestyle and CRC (99). Control subjects were more likely to be in the highest tertile of MDS compared to CRC patients (*P* < 0.001). Compared to “low” MDS scores (tertile 1), both “high” (tertile 3) and “medium” (tertile 2) MDS scores associated with decreased odds of developing CRC. Higher MD adherence mitigated the increased risk of CRC associated with obesity, diabetes, and high alcohol consumption. Conversely, physical activity (PA) did not seem to exert a protective effect.

A pooled analysis of three Italian case–control studies (*n* = 3,745 CRC cases, *n* = 6,804 controls) in populations across several Italian cities (100) concluded that all tertiles of MDS score above reference (MDS score = 0–2) were associated with a decreased risk [11%/unit increase in MDS score, (OR = 0.89, CI_95%_ = 0.86–0.91)] of CRC with a significant linear trend across all tertiles (*P*_trend_ < 0.0001). In secondary analyses stratified by cancer site, the MD decreased risk of colon, proximal colon, distal colon, and rectal cancer (100) (Table 4).

**Table 4:** Summary of studies investigating association between MD and site-specific cancer.

Cancer site	Reference	Effect	HR/OR/RR (95% CI)
**Colon**			

	Fung et al. (103)	No effect	RR = 0.89 (0.76–1.05)
		No effect (M)	RR = 0.87 (0.67–1.13)
		No effect (W)	RR = 0.91 (0.74–1.11)
	Agnoli et al. (24)	Decreased risk	HR = 0.54 (0.36–0.81)
	Bamia et al. (102)	Decreased risk	HR = 0.88 (0.78–1.00)
	Grosso et al. (99)	Decreased risk	OR = 0.48 (0.26–0.89)
	Rosato et al. (100)	Decreased risk	OR = 0.49 (0.39–0.60)

**Proximal colon**			

	Reedy et al. (101)	No effect (M)	RR = 0.83 (0.66–1.04)
		No effect (W)	RR = 0.84 (0.61–1.84)
	Agnoli et al. (24)	No effect	HR = 0.73 (0.33–1.61)
	Bamia et al. (102)	No effect	HR = 0.92 (0.76–1.11)
	Rosato et al. (100)	Decreased risk	OR = 0.48 (0.32–0.73)

**Distal colon**			

	Reedy et al. (101)	Decreased risk (M)	RR = 0.68 (0.53–0.86)
		No effect (W)	RR = 0.89 (0.76–1.84)
	Agnoli et al. (24)	Decreased risk	HR = 0.44 (0.26–0.75)
	Bamia et al. (102)	Decreased risk	HR = 0.83 (0.68–1.00)
	Rosato et al. (100)	Decreased risk	OR = 0.55 (0.41–0.74)

**Rectum**			

	Reedy et al. (101)	Decreased risk (M)	RR = 0.69 (0.54–0.88)
		No effect (W)	RR = 0.75 (0.50–1.21)
	Fung et al. (103)	No effect	RR = 0.78 (0.58–1.05)
		No effect (M)	RR = 0.75 (0.46–1.23)
		No effect (W)	RR = 0.80 (0.55–1.15)
	Agnoli et al. (24)	Decreased risk	HR = 0.41 (0.20–0.81)
	Bamia et al. (102)	No effect	HR = 0.90 (0.76–1.07)
	Grosso et al. (99)	Decreased risk	OR = 0.41 (0.18–0.95)
	Rosato et al. (100)	Decreased risk	OR = 0.58 (0.44–0.75)

##### Cohorts

Unlike case–control studies, which suggested a decreased risk of CRC with adherence to a MD, results of cohort studies (Table 3) have been somewhat inconclusive. For example, one study with the Italian cohort (age = 25–70 years) of the EPIC study reported a decreased risk of CRC in a pooled analysis with men and women (24). Another study reported protection for men in a cohort from the USA (mean age = 62.6 years) (101) and another reported it for women in the overall EPIC cohort (age = 25–70 years) (102). Conversely, data from the Nurse’s Health Study (NHS, women aged 30–55 years) and Health Professionals Follow-Up Study (HPFS, men aged 40–75 years) (103), and the Women’s Health Initiative (WHI) cohort study (women aged 50–79 years) (104) suggested no effects of the MD on CRC risk.

Protective effects of a MD eating pattern have been suggested for men in an analysis of the National Institutes of Health-AARP Diet and Health Study (NIH-AARP) (101). Investigators used data from a 124-item food-frequency questionnaire (FFQ) to explore the association between four dietary quality indexes (MDS), Healthy Eating Index (HEI)-2005, Alternate Healthy Eating Index (AHEI)-2005, and Recommended Food Score and CRC risk. Cox proportional hazard models adjusted for age, ethnicity, education, BMI, smoking, PA, TEI, and hormone replacement therapy (HRT) (women) indicated a decreased risk for men and no effect in women. The mean age of subjects in the first and fifth quantile of MDS adherence was, respectively, 61.5 and 62.6 years for men, and 61.4 and 62.1 years for women (101). When considering specific cancer sites, investigators reported no effect on risk of tumors of the proximal colon for both men and women. However, adherence to a MD decreased risk of distal colon and rectal cancer in men but not in women. Investigators attributed these sex-specific effects of the MD to differences between how men and women completed the FFQ in the NIH-AARP study, which may have led to increased measurement errors and non-differential biases among women. Other biases may have been a smaller sample size and fewer cases for women. Possibly, biochemical differences between men and women may influence the etiology of CRC and the response to a MD.

In support of this hypothesis, data from the EPIC cohort supported a decreased risk for women, but no benefit for men (102). About 58% of men and ~67% of women participants were below the age of 55 years, and both the MMDS and center specific (CS)-MMDS were applied to FFQ to establish MD adherence. Cox proportional hazard models adjusted for sex, age at enrollment, BMI, PA, education level, and smoking status revealed a decreased risk of CRC for all tertiles of MMDS and CS-MMDS scores with a significant linear trend for both scores (MMDS, *P*_trend_ = 0.02; CS-MMDS, *P*_trend_ = 0.05). For 2-unit increase in index score there was a 3% (MMDS) to 4% (CS-MMDS) reduction in CRC risk. However, neither association reached statistical significance (MMDS, HR = 0.96, CI_95%_ = 0.92–1.00; CS-MMDS, HR = 0.97, CI_95%_ = 0.93–1.01). There was no association between CRC risk and either score for men, whereas in women, there was a decreased risk associated with higher MMDS but not CS-MMDS. A decreased risk was reported for colon, distal colon, and rectal cancers but no association was observed for proximal colon tumors (102).

A study that applied the IMDI to data from the Italian cohort of the EPIC study (mean ~50 years of age) did not support a gender-specific effect of a MD pattern on CRC risk (24). Rather, higher IMDI was associated with a decreased risk in the overall cohort. Multivariate Cox proportional hazard models adjusted for non-alcoholic energy intake, gender, age, BMI, smoking, education and total PA revealed a decreased risk among all tertiles of IMDI above reference with a significant linear trend (*P*_trend_ = 0.043). Sex-specific analyses revealed a protective effect for both men and women when comparing the highest (6–11) to the lowest (0–1) score of IMDI. Further analyses indicated a decreased risk for colon, and distal colon but no association was reported for proximal colon and rectal cancers (24).

Results from the NHS (women aged 30–55 years) and HPFS (men aged 40–75 years) (103) studies did not support an association between MD and CRC risk. Data derived from FFQ were applied against the aMED and the Dietary Approaches to Stop Hypertension (DASH) diet indexes in Cox proportional hazard models adjusted for a number of variables including age, BMI, alcohol intake, family history, PA, aspirin use, colonoscopy, history of polyps, multivitamin use, smoking, and TEI. Investigators found no association between aMED score and risk of CRC in a pooled analysis of all subjects from both cohorts and in separate analyses for men (HPFS) and women (NHS). However, the DASH diet was protective in a pooled analysis (RR = 0.80, CI_95%_ = 0.70–0.91) and for women specifically (RR = 0.80, CI_95%_ = 0.67–0.94). There was no association between MD adherence and incidence of either colon or rectal cancer. The disparity in estimated cancer effect between the two MD indexes was attributed at least in part to differences in dietary components. Specifically, the DASH index considered low-fat dairy intake, which was inversely associated with CRC risk, whereas the aMED index did not. The DASH index may have also had more discriminating power by scoring each component on a 5-point scale, compared to the aMED, which scored on a 2-point scale. It is also worth noting that the population under study consisted of health care professionals, whose lifestyle and dietary habits may already be healthier than those of the general public.

Recently, Vargas et al. (104) found no association between MD adherence and CRC risk. The association between the aMED, DASH, HEI-2010, and AHEI-2010 indexes and CRC incidence was assessed in postmenopausal women (age 50–79 years) using data derived from the WHI study. Cox proportional hazard models adjusted for age, race/ethnicity, PA, educational level, smoking status, and HRT revealed no association between aMED score and CRC-incidence and -related mortality, whereas HEI-2010 and DASH indexes associated with a lower risk (HEI-2010: HR = 0.73, *P* < 0.01; DASH: HR = 0.78, *P* = 0.03). There were no overall differences in mean age between women in the fifth (63.5 years) and first (~63.0 years) quantile of aMED adherence.

We identified some studies that investigated the effect of MD on CRC-related mortality (Table 5). Data from the Multiethnic Cohort (MEC) (105) suggested a decreased risk of CRC-related mortality in women, with a 3% decrease in risk for every standard deviation unit increase in aMED score. There was also a decreased risk of all-cause mortality associated with aMED scores in women. There was no association between either CRC-related or all-cause mortality and aMED scores among men. After correcting for ethnicity, a higher aMED score associated with decreased risk only for African American women and for colon cancer specifically (105). Fung and colleagues (106) also investigated the association between various dietary quality indexes, including aMED, on CRC survival in women diagnosed with stages I–III CRC (*n* = 1,201). Investigators found that only a higher AHEI-2010 score significantly associated with lower overall mortality, but did not observe an association between aMED score and CRC-related mortality in a multivariate-adjusted model comparing highest to lowest quintile of MD score.

**Table 5:** Summary of studies investigating association between MD adherence and colorectal cancer-related mortality.

Reference	Cohort	Sex	Age	Model	Effect	HR/OR/RR (95% CI)	*P*-Value
Vargas et al. (104)	WHI	W	50–79	aMED	No effect	HR = 0.90 (0.57–1.43)	0.66
Jacobs et al. (105)	MEC	M/W	45–75	aMED	No effect (M) Decreased risk (W)	HR = 1.05 (0.81–1.28) HR = 0.88 (0.64–1.19)	>0.05 0.004
Fung et al. (106)	NHS	W	66.5^a^	aMED	No effect	HR = 0.87 (0.63–1.21)	0.31

*^a^Median*.

Overall, results of cohort studies suggest prevention of CRC by a MD may be influenced by scoring index; tumor site; and other variables related to type of population, country of origin, age, and gender. These variables deserve to be tested.

##### Meta-Analyses

Table 6 summarizes meta-analyses that investigated the association between MD adherence and CRC risk. A meta-analysis conducted by Schwingshackl and Hoffman (107) showed that the combined analysis of case–control and cohort studies decreased CRC risk by 14% (107). Similar benefits (~17% decreased risk) were confirmed in an updated and more recent analysis by the same authors (108). Another meta-analysis evaluated the influence of a MD eating pattern, without a restriction on fat intake, on CRC risk (109). Adherence to the MD was defined as a pattern that placed no restriction on total fat intake and included two or more of the following components: a high MUFA:SFA ratio; high fruit and vegetable intake; high consumption of legumes; high grain and cereal intake; moderate red wine consumption; moderate consumption of dairy products; and low consumption of meat and meat products with increased intake of fish. Pooled analyses of primary prevention cohort studies (*n* = 9) indicated the MD eating pattern reduced by ~9% CRC incidence (109).

**Table 6:** Summary of meta-analyses investigating the association between MD and colorectal cancer risk.

Reference	No. of studies (design)	Effect	RR (95% CI, *I*^2^)	*P*-Value
Schwingshackl and Hoffman (107)	2 (case–control)	Decreased risk	RR = 0.85 (0.78–0.94, 45%)	0.001
	5 (cohort)	Decreased risk	RR = 0.86 (0.76–0.97, 70%)	0.02
	7 (total)	Decreased risk	RR = 0.86 (0.80–0.93, 62%)	0.010

Schwingshackl and Hoffman (108)	4 (case–control)	Decreased risk	RR = 0.79 (0.67–0.93, 65%)	0.004
	3 (cohort)	Decreased risk	RR = 0.84 (0.75–0.94, 56%)	0.002
	7 (total)	Decreased risk	RR = 0.83 (0.76–0.89, 56%)	<0.00001

Bloomfield et al. (109)	9	Decreased risk	RR = 0.91 (0.84–0.98)	Not reported

#### Fruits and Vegetables

To our knowledge, only five studies have reported on the effects of fruit and vegetable intake on CRC risk in the context of a MD eating pattern (Table 7) (24, 100–102, 110). In detail, a case–control study sought to determine dietary factors associated with CRC among a low-risk population from Southern Italy (110). In a multivariate analysis adjusted for age, sex, education, smoking status, and modifications in diet in the previous 10 years, investigators found no association between either vegetable or fruit consumption and CRC risk when comparing quantiles of high intake (vegetable ≥329 g/day; fruit ≥482 g/day) to those of low intake (vegetable ≤236 g/day; fruit ≤307g/day). Another study investigated the association between individual components of the MDS and CRC risk in the NIH-AARP study and reported no association for higher intakes of both vegetables (≥1.85 cups/day for men; ≥1.87 cups/day for women) (1 cup = 236.6 g) and fruit (≥2.29 cups/day for men; ≥2.32 cups/day for women) in men and women (101). Similarly, no changes were observed in the prospective analyses of the Italian EPIC cohort and the overall EPIC cohort for both men and women when comparing the highest tertiles of intake to the lowest (24, 102). In the Italian EPIC cohort, the range of consumption for the third tertile of intake was 160.7–950.1 and 391.9–3790.5 g/day for vegetables and fruits, respectively (24). The range of consumption for the first tertile of intake was 0–96.6 g/day (vegetables) and 0–249.2 g/day (fruits). In the entire EPIC cohort, median consumption levels within the third tertiles of intake for vegetables and fruits were 331.0 and 384.8 g/day, respectively, whereas median levels for the first tertiles were 88.6 and 83.1 g/day, respectively (102). In contrast, vegetable and fruit intake were associated with decreased risk of CRC in a pooled analysis of three Italian case–control studies conducted by Rosato et al. (100). However, this analysis may have been somewhat skewed since it included nut intake with fruits to estimate adherence to a MD pattern based on MDS.

**Table 7:** Summary of studies investigating association between fruit and vegetable intake and CRC risk.

Reference	Cohort/population	Sex	Age	Model	Effect	HR/OR/RR (95%CI)
Centonze et al. (110)	Southern Italy	M/W	34–90	70-item FFQ	*Vegetables*
					No effect	RR = 0.51 (0.25–1.04)
					*Fruit*	
					No effect	RR = 1.02 (0.53–1.95)

Reedy et al. (101)	NIH-AARP	M/W	50–71	MDS	*Vegetables*
					No effect (M)	RR = 0.94 (0.86–1.03)
					No effect (W)	RR = 0.98 (0.5–1.12)
					*Fruit*
					No effect (M)	RR = 0.94 (0.86–1.03)
					No effect (W)	RR = 1.04 (0.90–1.19)

Agnoli et al. (24)	Italian EPIC cohort	M/W	25–70	IMDI	*Vegetables*
					No effect	HR = 0.89 (0.69–1.14)
					*Fruit*	
					No effect	HR = 0.87 (0.68–1.12)

Bamia et al. (102)	EPIC	M/W	25–70	MMDS	*Vegetables*
					No effect	HR = 0.98 (0.89–1.08)
					No effect (M)	HR = 0.91 (0.80–1.06)
					No effect (W)	HR = 1.02 (0.90–1.16)
					*Fruit*	
					No effect	HR = 1.03 (0.97–1.08)
					No effect (M)	HR = 1.02 (0.90–1.17)
					No effect (W)	HR = 1.01 (0.91–1.12)

Rosato et al. (100)	Various Italian cities	M/W	19–74	MDS	*Vegetables*	
					Decreased risk	OR = 0.69 (0.63–0.75)
					*Fruit*	OR = 0.79 (0.73–0.87)
					Decreased risk	

#### Fiber

A high fiber intake may exert protective effects against CRC associated with consumption of red and processed meat. For example, a study reported that subjects on a vegetarian diet (30 g fiber/day as non-starch polysaccharides) or a red meat and high fiber diet (420 g red meat + 30 g fiber/day), had significantly higher levels of the NOC-specific DNA adduct O(6)-carboxymethyl guanine compared to individuals on a high red meat diet with no fiber (420 g/day for 15 days) (76). Moreover, analyses of data derived from the Spanish cohort of the EPIC study indicated that consumption of pro-NOC forming compounds was lower in the MD group compared with groups adopting other dietary patterns (111).

Higher intake of fiber tends to accelerate transit time through the digestive system, thereby reducing exposure of the large intestine to potential carcinogens [i.e., secondary bile acids (BAs), NOC] (112). In support of a preventative role for fiber against CRC is a prospective analysis of data from the EPIC study. Remarkably, doubling fiber consumption in subjects with low intake reduced CRC by ~40% (113). Another study in men and women from the USA reported that subjects in the highest quintile of fiber intake had a 27% lower risk of developing adenomas, compared to subjects in the lowest quintile of intake (114).

#### Cereals, Whole Grains, and Legumes

Only a small number of studies examined the effects of cereals, grains, and legumes on CRC risk (Table 8). In a low CRC-risk population from Southern Italy, investigators found no protective effects of cereal intake when comparing individuals with higher (≥267 g/day) to lower (≤238 g/day) intake (110). In contrast, the NIH-AARP cohort study found that whole grain intake was associated with a decreased risk for men (≥1.19 oz/day), but not women (≥0.98 oz/day) (1 oz = 28.35 g) whereas legumes did not influence CRC risk for either men (≥0.09 cups/day) or women (≥0.06 cups/day) (101).

**Table 8:** Summary of studies investigating association between cereals, grains, and legume consumption and CRC risk.

Reference	Cohort/population	Sex	Age	Model	Effect	HR/OR/RR (95%CI)
Centonze et al. (110)	Southern Italy	M/W	34–90	70-item FFQ	*Cereals* and *grains*
					No effect	HR = 0.90 (0.45–1.80)

Reedy et al. (101)	NIH-AARP	M/W	50–71	MDS	*Cereals* and *grains*
					Decreased risk (M)	RR = 0.85 (0.78–0.93)
					No effect (W)	RR = 0.95 (0.83–1.08)
					*Legumes*
					No effect (M)	RR = 0.96 (0.88–1.05)
					No effect (W)	RR = 0.94 (0.83–1.08)

Agnoli et al. (24)	Italian EPIC cohort	M/W	25–70	IMDI	*Cereals* and *grains*
					No effect^a^	HR = 0.89 (0.70–1.15)
					*Legumes*
					No effect	HR = 0.86 (0.69–1.09)

Bamia et al. (102)	EPIC	M/W	25–70	MMDS	*Cereals* and *grains*
					No effect	HR = 0.96 (0.89–1.04)
					No effect (M)	HR = 0.96 (0.85–1.08)
					No effect (W)	HR = 0.92 (0.83–1.03)
					*Legumes*
					No effect	HR = 1.05 (0.95–1.17)
					No effect (M)	HR = 0.96 (0.81–1.13)
					Increased risk (W)	HR = 1.17 (1.01–1.34)

Rosato et al. (100)	Various Italian cities	M/W	19–74	MDS	*Legumes* Decreased risk	OR = 0.69 (0.64–0.76)

*^a^Pasta*.

In the Italian EPIC cohort, no association was found between pasta (used as proxy for grains) or legume intake and CRC risk when comparing the third (pasta, 71.9–431.5 g/day; legumes, 23.6–281.4 g/day) to the first (pasta, 0–37.9 g/day; legumes, 0–11.8 g/day) tertile of consumption (24). Cereal grain intake did not change CRC risk for the overall EPIC cohort, and in separate analyses for men and women when comparing the third (median = 309.9 g/day) to the first (122.1 g/day) tertile of intake (102). Legume intake (median 30.1 g/day) was reported to have no effect on CRC risk when compared to no consumption in the overall EPIC cohort and in men, whereas there was an increased risk reported in women with higher legume consumption (102). Rosato et al. (100) did not analyze cereal or grain intake in their study, however, they reported a decreased risk associated with legume intake.

#### Fish and Meat

Table 9 summarizes results of studies that investigated the association between fish and meat consumption and CRC risk. In a population from Southern Italy, there was no association between higher intake of fresh meat (88–131 g/day), beef (≥22 g/day), processed meat (≥3 g/day), and fish (≥51 g/day) (110). However, in the NIH-AARP cohort, increased consumption of red and processed meat (men, ≥3 oz/day; women, ≥1.8 oz/day) increased risk for women, whereas no association was observed for men (101). In the same study, fish consumption (men, ≥0.66 oz/day; women, ≥0.53 oz/day) had no effect in either men or women.

**Table 9:** Summary of studies investigating association between consumption of animal sources of protein and CRC risk.

Reference	Cohort/population	Sex	Age	Model	Effect	HR/OR/RR (95%CI)
Centonze et al. (110)	Southern Italy	M/W	34–90	70-item FFQ	*Meat*
					No effect (fresh meat)	RR = 0.74 (0.37–1.45)
					No effect (beef)	RR = 0.95 (0.50–1.80)
					No effect (processed)	RR = 1.01 (0.57–1.69)
					*Fish*
					No effect	RR = 1.07 (0.56–2.05)

Reedy et al. (101)	NIH-AARP	M/W	50–71	MDS	*Meat*
					No effect (M)	RR = 0.94 (0.86–1.03)
					Increased risk^a^ (W)	RR = 0.84 (0.74–0.96)
					*Fish*	
					No effect (M)	RR = 0.97 (0.89–1.06)
					No effect (W)	RR = 1.00 (0.88–1.14)

Agnoli et al. (24)	Italian EPIC cohort	M/W	25–70	IMDI	*Meat*
					No effect	HR = 0.94 (0.72–1.23)
					*Fish*
					No effect	HR = 0.88 (0.68–1.13)

Bamia et al. (102)	EPIC	M/W	25–70	MMDS	*Meat*
					No effect	HR = 1.08 (0.99–1.18)
					No effect (M)	HR = 1.07 (0.94–1.22)
					No effect (W)	HR = 1.08 (0.97–1.20)
					*Fish*
					Decreased risk	HR = 0.90 (0.82–0.99)
					Decreased risk (M)	HR = 0.85 (0.74–0.97)
					No effect (W)	HR = 0.94 (0.83–1.06)

Rosato et al. (100)	Various Italian cities	M/W	19––74	MDS	*Meat*	
					Increased risk^a^	OR = 0.86 (0.79–0.94)
					*Fish*	OR = 0.78 (0.71–0.85)
					Decreased risk	

*^a^Reverse scored comparing low intake to high intake, using high intake as referent*.

In the Italian EPIC cohort, higher intake of red meat (112–665.6 g/day) and fish (38.6–340.3 g/day) was reported to increase CRC risk compared to lower consumption (red meat, 0–69 g/day; fish, 0–20.1 g/day) (24). In the overall EPIC cohort, meat consumption did not change CRC risk, whereas fish consumption reduced risk when comparing the third tertile (median meat, 151.5 g/day; median fish, 63.8 g/day) to the first (meat, 43.9 g/day; fish, 8.6 g/day) tertile of intake (102). In the same study, the lack of association between meat consumption and CRC risk was confirmed for both men and women. Interestingly, a high intake of fish appeared to be protective only for men (102). In a pooled analysis of Italian case–control studies, an increased risk was reported for consumption of meat and meat products (100). In contrast, a decreased risk was reported for fish consumption.

Although red and processed meat have historically been considered to play a causative role in CRC development (115), we only identified two studies indicating an increased risk of CRC with higher meat consumption (100, 101). Red and processed meat have been classified by The World Health Organization and International Agency for the Research on Cancer (116, 117) as group 2A (“probably carcinogenic to humans”) and group 1 (“carcinogenic to humans”) carcinogens, respectively. Observational and mechanistic evidence from over 800 scientific articles support the recommendation from these agencies that intake of processed meat contributes to colorectal and gastric cancers. Turner and Lloyd (115) sought to further investigate the mechanistic link between red meat and CRC in a systematic review of forty studies using animal and cell culture models. These investigators concluded that most of the studies delivered meat or meat-derived compounds at doses that appeared to be significantly higher than those commonly present in the human diet. Interestingly, the presence of certain dietary compounds (i.e., chlorophyll, fermentable fiber, calcium carbonate, among others), fruits, and vegetables, and whole grains tended to mitigate, and in some instances even antagonize, the procarcinogenic effects of meat and meat-derived compounds. Possibly, adherence to a whole MD pattern may explain the reduced association between meat consumption alone and CRC risk in certain Mediterranean populations.

#### Fats

Key elements of the MD include reduced consumption of SFA from butter and higher intake of MUFA from olive oil. Although olive oil consumption is recommended at every meal in the MD eating pattern, only two studies have investigated its specific effect on CRC risk (24, 118) (Table 10). Data derived from case–control studies conducted throughout six geographical areas of Italy (118) indicated a decreased risk of CRC associated with olive oil intake when comparing higher intake levels (≥43.4 g/day) to lower (≤23.4 g/day). However, no association was reported for intake of butter ≥3.2 g/day compared to ≤1.0 g/day. Also, no association was reported for butter intake and colon or rectal cancer (118). In keeping with the latter results, analyses of the Italian cohort of the EPIC study found no association between higher intakes of either olive oil (29.9–160.4 g/day) or butter (1.4–101.1 g/day) and CRC risk (24). First tertiles of intake were 0–19.3 g/day for olive oil and 0–0.2 g/day for butter.

**Table 10:** Summary of studies investigating association between consumption of butter, olive oil, dairy, potatoes, sugar, and alcohol and CRC risk.

Reference	Cohort/population	Sex	Age	Model	Effect	HR/OR/RR (95%CI)
**Olive oil and butter**						

Braga et al. (118)	Various Italian locations	M/W	23–74	78-item FFQ	*Olive oil*	
					No effect	HR = 0.88 (0.68–1.14)
					*Butter*	
					No effect	HR = 1.00 (0.79–1.26)

Agnoli et al. (24)	Italian EPIC cohort	M/W	25–70	IMDI	*Olive oil*	
					Decreased risk	OR = 0.83 (0.70–0.99)
					*Butter*	
					No effect	OR = 0.93 (0.80–1.07)

**Dairy**						

Centonze et al. (110)	Southern Italy	M/W	34–90	70-item FFQ	Decreased risk	RR = 0.46 (0.22–0.98)

Bamia et al. (102)	EPIC	M/W	25–70	MMDS	Decreased risk	HR = 0.85 (0.78–0.92)
					Decreased risk (M)	HR = 0.88 (0.78–1.00)
					Decreased risk (W)	HR = 0.84 (0.75–0.93)

Rosato et al. (100)	Various Italian cities	M/W	19–74	MDS	Decreased risk	OR = 1.09 (1.00–1.19)

**Potatoes and sugar**						

Centonze et al. (110)	Southern Italy	M/W	34–90	70-item FFQ	*Potatoes*	
					No effect	HR = 1.12 (0.86–1.44)
					*Sugar*	
					Increased risk	RR = 2.75 (1.26–5.97)

Agnoli et al. (24)	Italian EPIC cohort	M/W	25–70	IMDI	*Potatoes*	
					No effect	RR = 0.80 (0.41–1.56)
					*Sugar*	
					No effect	HR = 0.98 (0.78–1.22)

**Alcohol**						

Reedy et al. (101)	NIH-AARP	M/W	50–71	MDS	No effect (M)	RR = 0.91 (0.82–1.00)
					No effect (W)	RR = 0.93 (0.78–1.02)

Agnoli et al. (24)	Italian EPIC cohort	M/W	25–70	IMDI	No effect	HR = 1.23 (0.96–1.58)

Bamia et al. (102)	EPIC	M/W	25–70	MMDS	No effect	HR = 1.05 (0.96–1.14)
					Increased risk (M)	HR = 1.20 (1.06–1.35)
					No effect (W)	HR = 0.98 (0.88–1.08)

Rosato et al. (100)	Various Italian cities	M/W	19–74	MDS	No effect	OR = 1.06 (0.96–1.18)

#### Dairy

The MD eating pattern recommends moderate consumption of low-fat dairy foods. Interestingly, cumulative evidence from three studies (Table 10) suggests that consumption of dairy products may actually be protective (100, 102, 110). For example, a study in a Southern Italian population (average 34–90 years) reported a decreased risk associated with higher (≥263 g/day) compared to lower (≤130 g/day) dairy consumption, which showed a significant linear trend across tertiles (*P*_trend_ = 0.05) (110). Similarly, a decreased risk of CRC associated with dairy intake was reported in a pooled analysis within the EPIC cohort when comparing the third (median = 529.8 g/day) to the first (median = 114.0 g/day) tertile of intake. Furthermore, a decreased risk was noted for both men and women in individual analyses by gender (102). Similar results were reported in the pooled analysis of Italian case–control studies, in which low consumption of dairy was associated with increased risk of CRC (100). Overall, these data suggest that the impact of dairy consumption in the context of a MD on CRC risk needs to be further examined to better define recommended intakes and potential complications associated with intake of SFA on cardiovascular diseases.

#### Potatoes and Sugars

Table 10 summarizes the results of studies that investigated the association between intake of sugars and potatoes on CRC risk. A 175% increase in risk of CRC was reported among individuals from Southern Italy (34–90 years) with the highest intake of foods with refined sugars (≥26 g/day), and a significant linear trend was observed across tertiles of intake (*P*_trend_ = 0.01) (110). This study however, reported no association between potato consumption (≥22 g/day) and CRC risk (110). Agnoli et al. (24) reported on soft drink consumption in the Italian cohort of the EPIC study, which we used here as a proxy for sugar intake. In a pooled analysis of men and women, no association was noted between higher consumption of soft drinks (14.4–3,000 g/day) and CRC risk when compared to no intake (24). Similarly, no association was found for higher intake levels of potatoes (34.7–420.9 g/day) (24).

#### Alcohol

Alcohol intake is considered a risk factor for CRC (119). Therefore, the recommendation for moderate alcohol consumption in the MD eating pattern has been called into question. A possible confounder when studying the association between alcohol intake and CRC risk is the type of alcohol consumed. Although the MD recommends moderate consumption of red wine, several studies looked at total alcohol consumption (Table 10). For example, one study that examined red wine intake in an unadjusted model reported no change in risk of CRC associated with low (0.25 l/day) consumption. However, medium (0.5 l/day) and high (>0.5 l/day) wine intakes were associated with increased risk compared to no consumption, and there was a significant linear trend across tertiles (*P*_trend_ = 0.006). On the other hand, using an adjusted model, no significant association was noted for “low” (tertile 2), “medium” (tertile 3), or “high” (tertile 4) intake (110). Similarly, analyses of data from the NIH-AARP study indicated no association between alcohol consumption (5–25 g/day) and CRC risk for either men or women (101).

A pooled analysis of men and women from the Italian cohort of the EPIC study, found no associated risk for any category of alcohol intake (24). Similar conclusions were reached in a pooled analysis of men and women from the overall EPIC cohort when comparing the third (median = 23.1 g/day) to the first (0.4 g/day) tertile of intake (102). However, separate analyses revealed both a gender- and dose-dependent effect. In men, there was no association with CRC risk for the middle tertile of alcohol intake (median intake = 5.6 g/day), whereas an increased risk was observed for the highest tertile (median = 23.1 g/day). In women, neither category of intake was associated with higher risk (102).

To evaluate the association between CRC and alcohol consumption (type and quantity), Kontou et al. conducted a case–control study with patients from the Saint Savvas Cancer Hospital and Alexandra General Hospital, Athens, Greece (119). Using an adjusted model, these investigators estimated that moderate alcohol intake (12–35 g/day) was associated with decreased CRC risk; higher levels (36–48 g/day) had no association, whereas the highest level of intake (> 48 g/day) was associated with increased risk (119). Individual analysis by gender indicated similar results for both men and women, except that the increased risk associated with intake >48 g/day was not significant for women.

In a case–control study involving Italian patients from Catania, Sicily, investigators examined the impact of adherence to a MD eating pattern and level of alcohol consumption (12–35, 36–48, and >48 g/day) on risk of CRC (99). For subjects with low or high adherence to the MD, alcohol consumption of 12–35 or 36–48 g/day was not associated with CRC risk. However, an increased risk was reported for subjects with low adherence to the MD and high alcohol consumption (>48 g/day), whereas at this level of alcohol intake there was no association in subjects with high adherence to the MD. These data suggested that following a MD eating pattern may mitigate some of the risk of CRC associated with higher alcohol intake (99).

Rosato et al. directly addressed the issue regarding alcohol quantity by calculating the associated odds ratio of “moderate” consumption using “high” and “no” consumption as reference quantiles (100). Moderate consumption was defined as consumption higher than zero but below or equal to the study- and sex-specific median values, whereas high consumption was defined as being above the median value. Based on these definitions, moderate consumption had no association with CRC risk compared to no or high consumption (100).

## Discussion

Adherence to a MD and consumption of certain compounds characteristic of this eating pattern may protect against the development of CRC and IBD. In general, case–control studies conducted in Mediterranean populations consistently supported decreased risk of CRC when using the MDS and MMDS scoring systems (73, 99, 100). On the other hand, there have been discrepancies in conclusions from cohort studies, with one study reporting a decreased risk in both sexes (24); two studies indicating no effect (103, 104); and two studies reporting sex-specific effects for men (101) and women (102). These inconsistencies across cohort studies may be related to the different scoring indexes utilized to determine MD adherence. For example, cohort studies that utilized the MDS, MMDS, and IMDI found a protective effect for men (101), women (102), and both sexes (24), respectively. In contrast, cohort studies employing the aMED failed to identify any protective effect associated with MD adherence (103, 104). The MDS and MMDS differ in that the MMDS accounts for PUFA in the lipid ratio. We found no case–control studies that utilized either the aMED or IMDI systems, which differ in regard to food categories (aMED and IMDI); accepted range of alcohol intake (aMED and IMDI); and method to determine point allocation (IMDI). In spite of these inconsistencies, results of observational studies and three meta-analyses available in PubMed point to a decreased risk of CRC associated with adherence to a MD. The results from the meta-analyses provide more robust estimates than those derived from individual studies (e.g., case–control, cohorts).

A decreased risk of CRC is generally reported for Mediterranean inhabitants that have traditionally followed a MD eating pattern (e.g., Italians, Greeks, EPIC cohort, Italian EPIC cohort) (24, 73, 99, 100, 102). Perhaps not surprisingly, studies reporting no association generally dealt with populations where the MD eating pattern was not as widely followed, such as the USA (NHS, HPFS, WHI) (103, 104). A determining factor in assessing adherence to, and efficacy of, a MD eating pattern may be differences in reference intake levels (e.g., quantiles) between Mediterranean and non-Mediterranean groups (i.e., NIH-AARP, NHS, WHI). This is an important consideration when interpreting results using indexes that distinguish levels of intake based on study-specific distributions, rather than predetermined absolute values. Thus, investigations conducted with Mediterranean populations (i.e., Italy, Greece, etc.) may have inherently higher cut off points for certain quantiles of intake than studies conducted with non-Mediterranean subjects (i.e., the USA). Furthermore, when MD indexes are applied against FFQ that assess intake over a relatively short period of time (previous 12 months), results may be skewed by confounding effects due to previous dietary practices. Therefore, the potential health benefits of adopting a MD pattern may not be as measurable for study participants that have adhered to undesirable dietary practices for the majority of their lifetime.

Age appears to be another determining factor in regard to the sex-specific effects of a MD. The study by Reedy and colleagues (101) and data from the WHI cohort (104) suggested no association between MD adherence and CRC risk in women of a relatively older age (~62 years). In contrast, data from the EPIC cohort suggested a decreased risk in women (102). However, the majority of women in the EPIC were younger, with ~67% of the subjects under the age of 55 years. In line with the hypothesis that a MD pattern has a greater impact in younger individuals are the results from the Italian EPIC cohort where the mean age of participants was ~50 years and MD adherence was reported to be protective for both sexes (24). These results suggest that lifetime adherence to a MD may exert beneficial effects not easily achievable in older groups. To complicate the interpretation of data related to interactions between age and MD adherence are results of one study (103) indicating that even in a relatively young population of women (NHS, 30–55 years of age) there was no association between aMED score and CRC risk. A possible explanation for the lack of an association in this study is the confounding effect imparted by the type of population analyzed, which consisted of health care professionals that likely already adhered to healthier lifestyles.

Overall, results from this review indicate the protective effect of the MD in regard to CRC risk may be specific to the distal colon. The majority of studies investigating proximal colon cancer (PCC) incidence reported no effect, except for the study of Rosato et al. that reported a decreased risk (100). On the other hand, all of the studies investigating distal colon cancer (DCC) noted a decreased risk with MD adherence. This disparity in the preventive effects of the MD on PCC versus DCC may be due to biological differences in the colonic mucosa of these regions, which are acquired *in utero* and in postnatal development, and may influenced epigenetically by environmental factors such as dietary exposures that occur later in life (120). There could also be differences in the presence of procarcinogenic factors between the ascending and descending colon (120).

### Frontiers in CRC Research

#### Epigenetics of CRC

Experimental evidence suggests an important role of epigenetic modifications in the development of CRC (121–123). Epigenetic modifications relate to changes in methylation of cytosine-guanine (CpG) dinucleotides (DNA methylation), histone-tail posttranslational modifications, and expression of non-coding RNAs (ncRNA) (123). To date, no studies have detailed the epigenome of individuals adhering to a MD eating pattern. In this section, we briefly review some of the epigenetic marks commonly observed in CRC. Then, we highlight some of the epigenetic modifications elicited by bioactive compounds commonly present in MD foods.

##### DNA Methylation

DNA methylation refers to the covalent attachment of a methyl group to the five position of a cytosine molecule within a CpG dinucleotide in DNA. These methylation reactions are catalyzed by DNA methyltransferases (DNMTs), of which three main isoforms exist: DNMT1, also known as the maintenance methyltransferase; and DNMT3a and 3b, which contribute to *de novo* methylation. The CIMP CRC subtype associates with a high frequency of CpG hypermethylation and is diagnosed based on the methylation status of various genes that participate in regulation of calcium transport (*CACNA1G*), proliferation (*IGF2*), Wnt signaling (*NEUROG1*), transcription activity (*RUNX3*), and suppression of cytokine signaling (124, 125). Hypermethylation of *MLH1* involved in DNA mismatch repair, and *TIMP3*, which inhibits metalloproteinases, also associate with the CIMP phenotype (126).

Approximately 12–17% of all CRC have MSI (123), of which 80% harbor silenced mismatch repair pathways as a result of biallelic hypermethylation of the *MLH1* gene. Loss of MLH1 expression potentiates replication errors in microsatellite sequences (123). Hypermethylation of genes involved in cell cycle regulation (*CDKN2A/p16/MTSI*) and repair of mutagenic DNA lesions (*MGMT*) contributes to formation of adenomatous polyps and progression through the adenoma–carcinoma sequence (127–134). Loss of *MGMT* is associated with G > A *KRAS* mutations commonly observed in CRC (133). DNA methylation also plays a role in CRC *via* silencing of genes associated with the β-catenin/Wnt (*APC, SFRP, CDX2, MCC*), p53 (*IGFBP7*) and cell cycle control (*CDKN2A*) pathways (123). Conversely, DNA hypomethylation has been suggested to play a role in CRC through activation of proto-oncogenes and CIN (135, 136). For example, hypomethylation of the transposable DNA element long interspersed nuclear element-1 has been shown to activate the *MET, RAB3IP*, and *CHRM3* proto-oncogenes in CRC metastases (136). Mirchev et al. characterized the association between DNA methylation status of the *MLH1, p16^INK^, TIMP3*, and *TPEF* genes and various clinicomorphological features of CRC (126). These investigators reported hypermethylation of *MLH1* and *p16^INK^* in elderly patients; *MLH1, p16^INK^*, and *TIMP3* in proximal tumors; and *p16^INK^* in poorly differentiated tumors.

The farnesoid receptor X (FXR) is a nuclear transcription factor that has been recognized as a tumor suppressor protein in intestinal mucosa. The FXR regulates BA homeostasis by controlling intestinal reabsorption, enterohepatic circulation, hepatic *de novo* synthesis, and intracellular regulation of BA (137–139). FXR deficiency results in enhanced tumor development in APC^Min/+^ mice (140), whereas adenoviral-mediated overexpression of constitutively active FXR in HT29 xenografts inhibits tumor growth *via* the induction of the proapoptotic *FAS, BAK1, p21, KLF4, FADD, CASP9*, and *p27* genes; and downregulation of antiapoptotic *BCL2* and proinflammatory *TNFα* (141). The tumor suppressor activity of FXR is likely related to both BA-dependent (i.e., protection from BA-mediated inflammation and toxicity) and BA-independent (i.e., gut permeability, Wnt/β-catenin signaling) functions (142). Bailey et al. investigated the regulation of FXR at various stages of CRC development by comparing polyp (*n* = 32) and adenocarcinoma tissue (stages I–IV, *n* = 43, 39, 68, and 9, respectively) to normal colon tissue (*n* = 238) (142). Compared to healthy colon tissue, FXR function and expression were decreased in polyps and precancerous lesions, whereas expression was silenced mostly at later tumor stages (I–IV). Data from the Cancer Genome Atlas revealed that ~12% of colon cancers have hypermethylated nuclear receptor subfamily 1, group H, member 4 (*NR1H4*) gene, which encodes for FXR (142). Interestingly, inhibition of DNMT1 activity with 5-azacytadine as well as silencing of *KRAS* with short-interfering RNA (siRNA) has been shown to significantly increase FXR expression (142). The latter data suggest the role of epigenetic mechanisms as regulators of FXR expression and susceptibility to CRC.

Recently, our laboratory reported that loss of *APC* predisposed to silencing of *FXR via* CpG hypermethylation in colonic mucosa of APC^Min/+^ mice and in HCT-116 human colon cancer cells (143). In APC^Min/+^ mice, CpG methylation of the FXR promoter was linked to decreased expression of FXR and ileal bile acid-binding protein (*IBABP*) and short heterodimer partner (*SHP*), two transcriptional targets of FXR; and increased expression of *COX-2*. In HCT-116 cells, siRNA-mediated knockdown of *APC* increased *c-MYC*, while decreasing FXR, expression. Treatment of APC knockout cells (HCT-116) with deoxycholic acid (DCA), a secondary BA, further reduced FXR expression. However, treatment of wild-type HCT-116, but not HT-29, cells with DCA induced FXR, which was associated with decreased promoter methylation. These cumulative results suggested that loss of APC function might favor epigenetic silencing of FXR, leading to decreased expression of factors involved in BA homeostasis (i.e., *IBABP, SHP*), and activation of others that contribute to inflammation (*COX-2*) and proliferation (*c-MYC*) (143). It remains largely unknown how food components that are common to the MD eating pattern alter epigenetically the APC-FXR axis. This hypothesis deserves to be tested.

Olive oil contains the phenolic compounds tyrosol, hydroxytyrosol, catechin, epicatechin, EGCG, oleuropein, quercetin, and rutin. In HT-29 colon cancer cells with inactivated APC, EGCG (20 µM) was shown to lower *p16^INK^* methylation after 6 days in cell culture conditions (144). Similar effects have been observed in RKO colon cancer cells treated with quercetin, which dose-dependently decreased *p16^INK^* promoter methylation and restored *p16^INK^* gene expression (145). The type 1 cannabinoid receptor (CB1) is a tumor suppressor encoded by *CNR1* with antiproliferative and proapoptotic activity in CRC cells (146). Loss of CB1 facilitates adenoma formation in APC^Min/+^ mice (147). In colon cancer Caco2 cells, EVOO and the individual compounds hydroxytyrosol and oleuropein were shown to increase *CNR1* and CB1 expression associated with decreased methylation of the *CNR1* gene (148). Rats given EVOO (10 days) had increased CB1 mRNA expression in colonic mucosa compared to controls. Overall, these observations suggest compounds commonly found in EVOO possess antagonistic properties against methylation of tumor suppressor genes.

The production of SCFAs, such as butyrate, acetate, and propionate through fermentation of fiber by gut microbiota may contribute to preventing the development of IBD and CRC (149). *In vitro* fermentation studies indicated that fiber sources commonly present in the MD support higher production of butyrate compared to fiber found in the Scandinavian dietary pattern (150). Preclinical studies demonstrated butyrate had anti-CRC activity associated with prevention of DNA methylation. For example, in LS174T colon cancer cells, butyrate decreased cell proliferation and rescued apoptosis-associated speck-like protein (ASC), a proapoptotic protein silenced by DNA methylation in CRC (151). In HT-29 colon cancer cells, butyrate was found to protect against genotoxicity induced by the secondary bile DCA (152), and lower DNMT1 levels (153). There was also evidence to suggest a synergistic effect between butyrate and DHA in regard to modulation of DNA methylation. In HCT-116 colon cancer cells, the combination of butyrate and DHA significantly reduced methylation of genes encoding the proapoptotic Bcl2l11, Cideb, Dapk1, Ltbr, and Tnfrsf2. Treatment with DHA alone also significantly reduced methylation of genes encoding Cideb, Dapk1, and Tnfrsf25, suggesting an association between *n*-3 fish oil and inhibition of DNMT activity (154).

A recent study (155) examined changes in methylation between baseline and 5 years postintervention in peripheral blood cells from patients (*n* = 36) in the PREDIMED-Navarra study. This study was a randomized, controlled, parallel trial with three groups of intervention in high cardiovascular risk volunteers, two with a MD and one low-fat control group. Eight genes related to inflammation and immune-competence (*EEF2, COL18A1, IL4I1, LEPR, PLAGL1, IFRD1, MAPKAPK2, PPARGC1B*) had changes in their DNA methylation levels that correlated with adherence to the MD. Interestingly, increased *EEF2* methylation levels positively correlated with reduced concentrations of inflammatory TNFα and CRP. These data support the idea that key components of the MD eating pattern (e.g., olive oil, fiber, fish oils) tend to exert protective, likely combinatorial, effects against DNA methylation changes associated with inflammation and CRC.

##### Histone Modifications

Histone posttranslational modifications (e.g., methylation, acetylation, ubiquitination, phosphorylation) influence compaction of chromatin and whether it is in an active or inactive state (123). For example, di- and tri-methylation of histone-3 (H3) lysine 4 (K4) (H3K4me2 and H3K4me3, respectively) and acetylation of H3 (H3Ac) and H4 (H4Ac) are commonly associated with relaxed chromatin and a transcriptionally permissive state. Conversely, tri-methylation of H3K9 and H3K27 (H3K9me3 and H3K27me3) are usually associated with chromatin compaction and transcriptional repression (156). Several examples of gene regulation *via* histone modification have been observed in the context of CRC. Wnt family member 5A (Wnt5a), a factor involved in regulation of the Wnt pathway, was found to be downregulated in metastatic CRC. In the SW620 human metastatic CRC cell line, downregulation of Wnt5a was linked to enrichment of H3K27me3 in addition to decreased levels of H3K4me2, and loss of H3Ac and H4Ac (157). The increase of H3Ac, H4Ac and H3K4me2 after butyrate treatment in SW620 cells confirmed the involvement of histone modifications in the transcriptional regulation of *Wnt5a*. Deacetylation of H3K9 was associated with loss of the calcium-sensing receptor in CRC (158). In HCT116 human colon cancer cells, the deleted in colon cancer (*DCC*) gene sequence was enriched with the repressive marks H3K9me3 and H3K27me3, whereas the permissive mark H3K4me3 was absent (159). Studies in HCT116 and SW480 CRC cell lines reported decreased levels of H3K4me3 and increased H3K27 associated with downregulation of mucin-like protocadherin (160). Based on these observations, monitoring of histone posttranslational modifications may prove useful hints as prognostic biomarkers of efficacy for the MD eating pattern against CRC. For example, histone trimethylation at H3K4, H3K9 and H4K20 was reported to be associated with better prognosis in early-stage CRC patients in regard to overall survival and tumor recurrence (161).

Butyrate is widely recognized as a histone deacetylase (HDAC) inhibitor (162). In HT-29 colon cancer cells, butyrate prevented TNFα-mediated activation of COX-2 transcription and protein synthesis similar to trichostatin-A, a synthetic high-affinity HDAC inhibitor (163). In HCT-116 cells, butyrate significantly increased global H3Ac compared to untreated cells (154). In RKO, HCT-116, and HT-29 CRC cells, sodium butyrate (5 mM) was found to induce apoptosis and cell cycle arrest, which were linked to inhibition of HDAC activity and decreased HDAC1, DNMT1, and surviving protein (153). Interestingly, the epigenetic effects of butyrate may be dependent on cellular energetics (164). In normal colonocytes, butyrate stimulates cell growth due to its preferential utilization as a fuel source *via* mitochondrial oxidative phosphorylation (164). However, in malignant cells, cellular energetics are deregulated in a phenomenon known as the Warburg effect, a phenotype that is characterized by increased utilization of glucose and glycolytic metabolism and decreased mitochondrial oxidation (165, 166). As a result of downregulated mitochondrial metabolism, butyrate may accumulate and exert epigenetic modulation (164). These data further highlight the potential role of fiber common to the MD as a preventative of CRC.

##### Non-Coding RNAs

Non-coding RNA refers to RNA transcripts that are not translated into proteins, and are usually classified into two groups. Short ncRNA (<30 nucleotides) include microRNAs (miRNAs), siRNAs, and piwi-interacting RNAs. Long ncRNA (>300 nucleotides) include the long intergenic ncRNA which target specific loci to regulate expression (167). Other examples of ncRNA include transfer RNA and ribosomal RNA. The best classified ncRNAs in regard to cancer development are miRs, which negatively regulate gene expression at the posttranscriptional level by either signaling the destruction of mRNA transcripts or blocking their translation into proteins (168). Examples of aberrant miR activity have been reported in both the traditional “adenoma–carcinoma” and “serrated” model of CRC. For instance, in the traditional model, dysregulation of Wnt/β-catenin signaling was associated with the miR-17-92a cluster, miR-135b, miR-143, and miR-145; genes involved in the RAS/MAPK were regulated by miR-143, let-7, miR-21, and miR-31; genes involved in the Pi3K/Akt pathway appeared to be controlled by miR-1, miR-21, and miR-143; and p53 was regulated by miR-34a/b/c, miR-133a, miR-143, and miR-145 (123).

Butyrate has been found to inhibit expression of the proliferative miR-92a in HCT-116 cells by decreasing expression of c-Myc, leading to increased rates of apoptosis (169). Another study in HCT-116 cells showed that butyrate treatment modulated the expression of 44 miRs including members of the miR-106b family involved in p21 regulation (170). In HT-29 cells, a combination of quercetin and resveratrol, polyphenolic compounds commonly found in the MD, decreased expression of oncogenic miR-27a; induced caspase activation; and decreased ROS formation (171). In rats, an EVOO-enriched diet was found to lower the expression of miR-23a and miR-301a (~50%) compared to control-fed animals (148). Fish oil was shown to protect against azoxymethane-induced miRNA deregulation in an AOM rat model (172).

Turning to the effects of a whole MD, a recent study investigated the influence of an eight-week MD eating pattern for weight loss [the RESMENA (reduction of MetS in Navarra, Spain) diet] on expression of inflammation-related miRNA and genes in white blood cells (WBCs) from individuals (age = 48.84 ± 10.02 years; BMI = 35.41 ± 4.42) with MetS. The expression of miR-155-3p was decreased in WBC, whereas Let-7b was strongly upregulated as a consequence of dietary intervention. Given the known repressive functions of members of the Let-7 family against cellular processes associated with cancer, the latter results highlighted the concept that a MD eating pattern may protect against CRC in particular in overweight or obese individuals (173).

Taken together, aberrant epigenetic processes play a major role in CRC development and progression. Given the myriad of bioactive compounds found in the MD eating pattern and the accumulating evidence suggesting epigenetic effects of such compounds, future investigations should characterize the epigenome, and subsequent down-stream phenotypes, of subjects adhering to a MD. Possibly, the protective role of the MD against CRC could be due to the sum of epigenetic modifications elicited by a combination of bioactive food components characteristic of this dietary patter.

### Microbiome and CRC

At the time of this writing, only a few investigations have focused on the relationship between MD adherence and changes in gut microbiota. Recently, the gut microbiome has emerged as a key factor in both the prevention and etiology of CRC (174), In an analysis of 153 individuals habitually following omnivore, vegetarian or vegan diets, identification of a MD pattern was associated with beneficial changes in the host microbiome including increased SCFA production and abundance of *Prevotella* and fiber-degrading *Firmicutes* (175). Conversely, lower adherence to the MD was associated with increased urinary trimethylamine levels, which has been linked to abundance of L-*Ruminococcus*, a bug commonly linked to CD susceptibility in mice. A clinical trial in healthy male volunteers indicated that daily intake of red wine polyphenols for 4 weeks significantly increased the number of *Enterococcus, Prevotella, Bacteroides, Bifidobacterium, Bacteroides uniformis, Eggerthella lenta, and Blautia coccoides-Eubacterium rectale*, as indicated by analysis of fecal samples (176). Fruit intake has been associated with increased *Bifidobacteria* and *Lactobacilli*, which are considered beneficial to the host (177). Intake of fruits and vegetables, in addition to flavonoid intake, were negatively associated with *Clostridium histolyticum/perfringens* groups, whereas starch intake was associated with total bacterial number and fiber consumption was positively correlated with *Bacteroides/Prevotella* (178). In C57BL/6J mice, olive oil supplementation has been associated with growth of members of the *Bacteroidaceae* family compared to mice supplemented with palm oil or given a mixture of flaxseed and fish oil (179).

In rats consuming a diet with protein contributing 20% of TEI, meat protein intake (from beef, pork, or fish) was associated with higher relative abundance of *Lactobacillus*, which was generally considered beneficial, but lower relative abundance of SCFA and SCFA-producing bacteria including *Fusobacterium*, and *Prevotella*, compared to soy-protein fed rats (180). Additionally, an association has been identified between red meat consumption and enrichment of *Bacteroides massiliensis, Alistipes finegoldii*, and *Bilophila wadsworthia* bacteria, which have been implicated in CRC etiology (181). Increased *Bacteroides*, which has been observed in CRC, was also associated with increased consumption of animal protein and saturated fat. The latter are characteristic of a Western style diet pattern (181).

## Conclusion

We have summarized evidence suggesting the MD may reduce CRC risk by affecting known biological factors associated with inflammation, genetic and epigenetic processes, and the host microbiome. However, it should be noted that observed changes in biomarkers may not necessarily translate to an improved clinical outcome. This is reflected in the disparity of health effects we observed among the observational studies. Case–control studies of the MD consistently reported a decreased risk of CRC, whereas results of cohort studies were somewhat inconsistent. Sources of variation include among others study design, age, ethnicity, and gender of the population under investigation. Studies that looked at the effects of the MD on site-specific cancers concluded that the decrease in tumor risk may be more pronounced for the distal colon as opposed to the proximal colon. However, results of meta-analyses support the adoption of a MD to decrease risk of CRC irrespective of tumor site. Additionally, other well-established benefits of the MD eating pattern (diabetes prevention; improved cardiovascular health, cognitive, and bone health, etc.) support its adoption as a healthy dietary choice. Suggested new frontiers of investigation include the role of the MD pattern on epigenetic regulation of gene expression and changes in microbiome associated with initiation and progression of CRC.

## Author Contributions

MD, OS, TD, and DR contributed to the conceptual development, organization, and review of the manuscript; MD and DR were responsible for systematic review of the literature, collecting and cataloging of published data, and writing of the manuscript; DR was responsible for the content of the manuscript.

## Conflict of Interest Statement

The authors declare that the research was conducted in the absence of any commercial or financial relationships that could be construed as a potential conflict of interest.
