# Supplementary material for: Mediterranean Diet: Prevention of Colorectal Cancer
Source: Front Nutr. 2017 Dec 5;4:59. doi: 10.3389/fnut.2017.00059 (PMC5723389; doi:10.3389/fnut.2017.00059)
Supplement: Supplementary file 1 [file Data_Sheet_1.PDF]

Supplemental Material 1

When using the MDS, participants are assigned a score of "1" or "0" for each food component based on intake. For the categories of cereals, fish, fruits and nuts, legumes, MUFA/SFA, and vegetables, a score of "1" is given for intake greater than or equal to the study-specific median, whereas a score of "0" is given for all other consumption levels. For the dairy and meat categories, subjects are given a score of "1" if their intake is below the median and a score of "0" for all other consumption levels. For alcohol consumption, one point is assigned for intakes of 5-25 g/d for women and 10-50 g/d for men.

aMED: categories are scored similarly to the MDS; intake of items indicative of the MD (fish, fruit, legumes, MUFA/SFA, nuts, vegetables, and whole grains) greater than or equal to the study-specific median is scored as "1" with lower levels of intake scored as "0". Intake of red and processed meat below the median is also scored as "1". Alcohol intake is scored as "1" for consumption of 5-15 g/d for both men and women. The aMED scoring range is also from 0-9, similar to the MDS. The aMED index differs from the MDS in that potatoes are excluded from the vegetable group; fruits and nuts are separated into distinct categories; the dairy group is eliminated; the cereals group is expanded to include all whole grains; only red and processed meat are considered in the meat category; and the range for moderate alcohol intake is lowered.

Italian MD index (IMDI): subjects are awarded "1" point if consumption of a food from the MD (fish, fruit, legumes, Mediterranean vegetables, olive oil, and pasta) is within the 3rd tertile of the study-specific distribution and "0" points for lower intake levels. Consumption of foods not within the MD eating pattern (butter, potatoes, red meat, and soft drinks) is reversed-scored, i.e. intake within the 3rd tertile is assigned a "0" and lower levels of consumption are given a score of "1". Alcohol consumption is given a score of "1" for intake up to 12 g/d, whereas no intake or consumption >12 g/d are scored as "0". This system differs from the MDS not only in the food components analyzed, but also how scores of 1 or 0 are distinguished. Whereas the MDS scoring is based on study-specific median intake, the IMDI is based on whether or not intake levels fall within the 3rd tertile of the study specific distribution.

A value of "0" is applied when consumption of healthy foods is below median intake levels, whereas a value of "1" is given when their intake is above the median. The consumption of detrimental foods below median intake levels is scored as "1", whereas their consumption above the median is scored as "0". For ethanol intake, a value of "1" is assigned to men and women with consumption between ~10-50 g/d and 5-25 g/d, respectively.

The modified MDS system (MMDS) varies from the MDS in that the lipid ratio includes PUFA in addition to MUFA against SFA. The food components assessed and the scoring procedure is the same as in MDS.
